# The association between paternal labour migration and the growth of the left-behind children—evidence from a birth cohort in Dhanusha district, Nepal

**DOI:** 10.1136/bmjgh-2025-021253

**Published:** 2026-01-09

**Authors:** Laura Busert-Sebela, Mario Cortina-Borja, Jonathan Wells, Delan Devakumar, Simon Eaton, Dharma S Manandhar, Shyam Sundar Yadav, Naomi M Saville

**Affiliations:** 1 University College London Great Ormond Street Institute of Child Health, London, UK; 2 Faculty of Life Sciences: Food, Nutrition and Health, Universität Bayreuth, Bayreuth, Germany; 3 Institute for Global Health, University College London, London, UK; 4 Mother and Infant Research Activities (MIRA), Kathmandu, Nepal

**Keywords:** Stunting, Nepal, Cohort study

## Abstract

**Introduction:**

We aimed to determine the association between paternal labour migration and the growth of the left-behind children in Dhanusha district, Nepal, where child stunting and international labour migration are highly prevalent.

**Methods:**

We used growth data at birth, 6 months, 1 year and 2 years from a birth cohort study conducted 2012–2014, and growth data at age 6 years collected in 2018. We collected household migration history data to determine the children’s exposure to paternal migration. The primary outcome was child length/height-for-age *z*-score (HAZ). Children’s body circumferences, skinfold thicknesses, body composition, tibia length and grip strength were secondary outcomes measured at 6 years. We tested (i) the overall association between paternal international migration and the growth of the left-behind child; the roles of (ii) the duration of migration (≤12 mvs >12 m) and (iii) child age (≤6 mvs 12–72 m) as moderating factors; (iv) the association between receipt of remittances from the migrant father and child growth outcomes; and (v) stratified the main analyses by child gender. We fitted mixed-effects linear regression models for longitudinal data and linear regression models for cross-sectional data, adjusted for potential confounders.

**Results:**

Analysing across all time points, daughters of labour migrants had lower HAZ than daughters of non-migrants (−0.13, 95% CI −0.24 to –0.03), but no overall association was found in boys. The negative associations were largest at <6 m (girls: −0.23, 95% CI −0.41 to –0.05), but in boys only if the father had recently (≤12 m) migrated (−0.26, 95% CI −0.51 to 0.00). Children of migrants showed a tendency towards smaller body sizes compared with children of non-migrants. We found no association between remittances and any measure of child growth.

**Conclusions:**

Interventions should target support for pregnant women and mothers with young infants to provide gender-equitable childcare, especially if their husband just left for work overseas.

**WHAT IS ALREADY KNOWN ON THIS TOPIC**

- Global evidence shows that left-behind children have shorter height for their age than children whose parents did not migrate, but most studies are from China and are on internal migration.

**WHAT THIS STUDY ADDS**

- In a labour-sending and remittance-dependent population of Nepal, we found no evidence of a positive association between paternal migration and the growth of left-behind children. At a very young age and shortly after the father left for work overseas, we observed a negative association with child growth, especially in girls.

**HOW THIS STUDY MIGHT AFFECT RESEARCH, PRACTICE OR POLICY**

- Labour migration policies need to be adjusted to better serve the interests of migrants and their families. Left-behind mothers and children require support, especially in the first months of life and soon after the father has migrated. Programmes should seek to address gender-discriminatory childcare practices.

## Introduction

 The majority of the global migrant population are labour migrants,^1^ many of whom leave behind children in their home communities. While there are no global estimates on the number of so-called ‘left-behind children’, it is likely to be in the hundreds of millions. In Nepal, 20% of children under 18 years have at least one parent (mostly fathers) living abroad.^2^

The remittances that labour migrants send back home are vital sources of income for their families and for the economies in their home countries. Nepal is a remittance-dependent country, where remittances made up 25% of the gross domestic product in 2023.^3^

Parents’ decisions to seek work abroad and leave the child(ren) behind are likely motivated by the hope that the additional income may improve their child(ren)’s well-being and prospects.^4^ However, a parent’s migration can potentially affect the left-behind child in both positive and negative ways, depending on circumstantial factors such as the age of the child, the duration of migration, whether the mother, father or both go abroad and who takes care of them in their absence and the costs and remittances of migration and how these are spent. In Nepal, the labour migration sector has been under scrutiny because of frequent instances of deception and exploitation of labour migrants.^5^ In the host countries, labour migrants face living and working conditions that put their health and lives at risk.^6^,^8^ Malpractice among recruiters at home and employers overseas makes the labour migration riskier and may limit the benefits for migrants and their families.

The aim of this study was to determine how the father’s international labour migration is associated with the growth of their left-behind child. Linear growth is a potent marker of child overall well-being and the extent to which the child’s needs for overall care, adequate nutrition, stimulation and hygiene are met.^9^ Low height-for-age is indicative of circumstances that prevent children from thriving and developing to their full potential.^10^ Furthermore, we considered additional growth indicators such as body circumferences, skinfold thickness and body composition as secondary outcomes. They have rarely been addressed in this context and can provide new nuanced insights into how children’s growth variability may be affected by fathers’ migration.

This study was conducted in Dhanusha district, an area of Nepal with high labour migration rates to Gulf countries, Malaysia and to a lesser extent to India. It is located in Province 2, which has one of the highest rates of child undernutrition in the country, with stunting prevalence at 34%.^2^

### Research questions and hypotheses

We addressed the following research questions (RQs) and hypotheses:

RQ 1: Is there a difference between the linear growth of children of labour migrants and non-migrant fathers?

Fellmeth’s 2018 systematic review suggests a small, negative association between parental labour migration and child growth. However, most studies included in this review were from China, where not just fathers but also mothers or even both parents migrate and leave their children while they move to the cities within the country for work. In our study population, usually only fathers go overseas while the mothers remain at home, often within extended families. Since the children’s main carers stay with them, the potential negative impact on the children may be smaller and children may benefit from increased household income through remittances. Hence, we hypothesised a net benefit of father’s migration on child growth:

Hypothesis 1: Children of migrant fathers are taller for their age than children of non-migrants.

RQ 2: Is there an association between the duration that the migrant fathers have spent abroad and the linear growth of the children?

Sending a household member overseas requires substantial investment. Most men finance their migration through loans from traditional moneylenders with high interest rates, typically 36%.^11^ Although most migrants send money home, it takes time until they can remit because they need to get settled in the destination country and earn enough money to send some back home. In this population, migrants typically send the first remittances 4 months after their departure. It takes time to pay back the large investments made and start profiting from earnings. In this population, one migration stint has a median duration of 2 years, after which migrants often come home for a short while and then apply again for a job abroad. Many migrants also choose to extend their stay abroad and only irregularly, if ever, visit their family during holidays. Moreover, the father’s departure can initially have a disruptive effect on the family. Hence, we hypothesised the following associations between the duration of migration and child growth.

Hypothesis 2a: Shortly after the fathers’ going abroad, the children’s growth is negatively affected such that children of migrants are smaller than children of non-migrants.

Hypothesis 2b: Children of longer-term migrants grow better than children of recent migrants or non-migrants.

RQ 3: Is there an age-period when children’s linear growth is more sensitive to the impact of fathers’ migration?

In the first 6 months of life, children are ideally exclusively breastfed and rely on their mother’s care to thrive, and we assume that the father’s migration does not affect breastfeeding practices or quality of maternal care. Once the children are introduced to complementary foods, they need a nutritious and diverse diet, so their growth might be improved by increased affordability of high-quality foods associated with remittance income.

Hypothesis 3: The association between fathers’ migration and child growth is larger in the complementary feeding period compared with the exclusive breastfeeding period.

RQ 4a: Is there an association between the fathers’ migration and other measures of child growth (body circumferences, skinfold thickness, body composition, tibia length) and function (grip strength)?

RQ 4b: Does the association differ by the timing of the migration relative to the children’s life?

The presence of the mother may protect the child from potential negative effects of paternal labour migration, and the increase in family income, especially after the exclusive breastfeeding period, may benefit the child.

Hypothesis 4a: Children of migrants have larger body circumferences, skinfold thickness, fat and lean mass, longer tibia and higher grip strength compared with children of non-migrants.

Hypothesis 4b: The association between father’s migration and these measures of child growth is larger in the complementary feeding period compared with the exclusive breastfeeding period.

RQ 5: Is there an association between the amount remitted and child HAZ and other measures of child growth (body circumferences, skinfold thickness, body composition, tibia length) and function (grip strength)?

We hypothesised that the remittances are used for purchase of nutritious foods and/or healthcare, benefitting the left-behind children.

Hypothesis 5: There is a positive association between the amount remitted and child growth in terms of HAZ and other growth outcomes.

## Methods

### Study design

This study builds on a longitudinal Growth Monitoring Study (GMS, 2012–2014) among children 0 to 24 months (n=602) in 60 clusters of Dhanusha district in the plains of Nepal. The details of the GMS are published elsewhere.^12^ Briefly, infants were enrolled within 72 hours of birth and followed up every 28 days until they were 2 years, with the aim of identifying the determinants of infant growth in this population. We found that maternal factors related to both the environment in utero and in postnatal life were the most important factors. The overall most important determinant of growth was low birth weight, followed by maternal education.

For the present study, we followed up this cohort in 2018 when the children were 6 years old. We took anthropometric measurements of children and recorded the household migration history.

### Outcomes

We extracted infant length-for-age *z*-score (LAZ) at birth, 6 months, 1 year and 2 years of age from the original GMS (2012–2014). At the follow-up in 2018 we measured their standing height. Infant supine length (0–2 years) and child height (6 years) were measured in duplicate by trained data collectors using a ShorrBoard stadiometer (Maryland, USA) accurate to 1 mm. We used the 2006 WHO growth standards^13^ to calculate infant LAZ and the 2007 WHO growth references^14^ to calculate child HAZ. Hereafter, we refer to both LAZ and HAZ as ‘HAZ’.

Body circumferences (head, mid-upper arm (MUAC), waist, hip), skinfold thickness (biceps, triceps, suprailiac, subscapular), body composition (lean mass (kg), fat mass (kg), fat mass index (kg/m^2^), lean mass index (kg/m^2^), lean mass *z*-score, fat mass *z*-score (using UK reference data,^15^ tibia length and grip strength (kg) were only measured at 6 years in 2018. All measurements were taken by experienced data collectors who had participated in training and standardisation sessions. All measurements were taken in duplicate, except skinfold thickness and body composition which were taken in triplicate. The Seca 212 head circumference tape was used to measure head, mid-upper-arm, mid-thigh and calf circumference. For waist and hip circumference, an insertion tape was used. Skinfold thickness was measured using callipers on the participants’ left side. Body composition (lean mass and fat mass) was measured using bioelectric impedance analysis (BIA, Bodystat 500 instrumentation) and a prediction equation (lean mass (kg)=2.730 + 0.788 height (m)^2^/impedance (ohm)) derived from an isotope calibration sub-study (n=54) which is described in detail in online supplemental material section 1. Fat mass was calculated as the difference between body weight and lean mass. Fat mass index and lean mass index are height-adjusted outcomes. Grip strength was measured using a digital dynamometer. All outcomes were continuous except for grip strength. Children who did not manage to pass the grip strength threshold of 5 kg had no value displayed on the dynamometer, so we used a categorical variable with one category for grip strength less than 5 kg.

### Exposures

#### Father’s migration and duration abroad

In the 2018 questionnaire, respondents recalled the year and month that the father left for/returned from international labour migration over a recall period of 7 years. We used these dates to determine the children’s exposure to their fathers’ migration in the periods preceding the time points birth, 6 months, 1 year, 2 years and 6 years. We also calculated the duration of migration up to these time points. A detailed description of how duration of migration was calculated can be found in online supplemental material section 2.

To address RQ2, we differentiated between short-term (1–12 months) and long-term (> 12 months) migrant fathers and included one category for non-migrant fathers.

#### Fathers’ net remittances

Most interviewees could provide a lump sum of remittances over the migrant’s whole migration cycle but not the amount and frequency of remittances. This meant it was not possible to link remittances to specific child ages. We could only calculate the cumulative amount remitted over the child’s lifetime. International labour migration is a substantial investment and there is considerable variability in the total costs of migration. We therefore decided to calculate the *net* remittances by summing the father’s total costs of migration across all migration cycles recorded over the recall period and subtracting it from the total amount remitted in that same time period.

### Potential confounding factors

Potential confounding factors in the relationship between the respective exposure and child growth were identified using Directed Acyclic Graphs (DAGs)^16^ and the software DAGitty^17^ (online supplemental material section 3). In all regression models, we adjusted for father’s education, asset quartile, maternal height and migration of other household members. When testing associations with fathers’ migration (RQs 1–4), we additionally adjusted for household food insecurity.

#### Socio-economic status

We used principal component analysis to generate asset scores and wealth quartiles at baseline (childbirth) and the last follow-up (6 years). We adjusted for maternal height as an indicator of the mother’s early life deprivation and long-term economic position, and to adjust for the genetic component of growth.

#### Paternal education

The remittances a father sends home depend on the destination country, the job that he obtains and most importantly the salary and benefits such as employer-provided housing. A potential migrant with higher education and better financial resources is more likely to access trustworthy brokers and negotiate a better contract. We included fathers’ education as a categorical variable: No education, primary to lower secondary education (class 1–8), secondary and above (class≥9).

#### Household food insecurity

We assessed food insecurity in the preceding 30 days at baseline and 6 years using the FANTA Household Food Insecurity Access category.^18^ Since few households suffered moderate/severe food insecurity, we classified households as food secure or suffering from any level of food insecurity.

#### Migration of another household member

If another household member is already overseas, he may be able to lower the costs of migration for the father by sharing information on good jobs, cheap remittance channels and economical living overseas. To determine the exposure to another household member’s migration, we followed the same procedure as with the father’s migration.

### Regression analysis

RQs 1–3: We fitted HAZ as an outcome using mixed-effects linear regression models (R package nlme^19^ to account for repeated measurements within children. We treated the child as the clustering unit (random-effects grouping factor). A natural cubic spline (2 df) for child age captured a flexible, non-linear growth trajectory. We assessed multicollinearity using adjusted generalised variance inflation factors (GVIF) and the car package.^20^ To address RQ3 (differences by child age), we ran separate analyses for children aged 0–6 months and 12–72 months. Models fitted over the whole age range (0–72 months) and for older children (12–72 months) had random effects on both intercept and slope, models fitted for younger children (0–6 months) had random effects on only the intercept. We additionally stratified our analysis by child gender.

RQ 4: We fitted linear regression models to estimate the relationship between father’s migration and body circumferences, skinfold thickness, body composition and tibia length at 6 years. For the categorical outcome grip strength, we fitted ordered logistic regression models.

RQ 5: We fitted linear regression to estimate the relationship between the fathers’ cumulative net remittances over the children’s lifetime and child HAZ, body circumferences, skinfold thickness, body composition, tibia length, and ordinal logistic regression for grip strength at 6 years.

For RQs 4 and 5, we did not stratify our analyses by child gender. The sample size and precision of estimates were already limited and would have been further compromised by additional stratification.

All analyses were conducted using R 4.4.2 and RStudio. The data and R code that support the findings of this study are openly available in the University College London (UCL) Data Repository at https://doi.org/10.5522/04/30969145.^21^

### Patients and public involvement

Patients/the public were not involved in this study.

## Results

### Descriptive results

**Figure 1:**
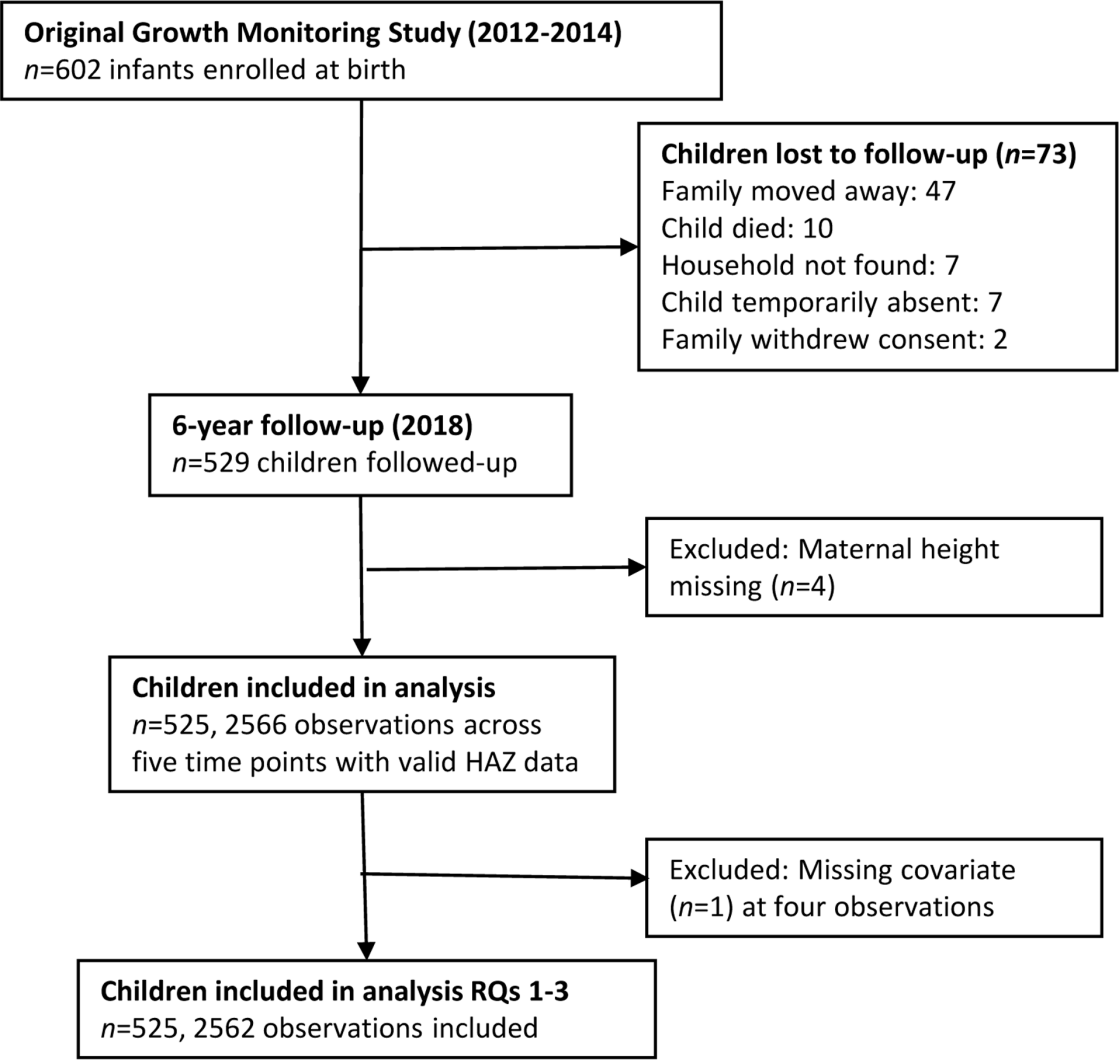
Study flow chart. HAZ, height-for-age *z*-score; RQs, research questions.

Migration history was only available for children followed up at 6 years (n=529, 89% of the original cohort who were still alive). Of these, four had missing maternal height and were excluded, so the final analytical sample included 525 children (figure 1). There was a higher number of missing data on body composition due to faulty BIA cables (n=37) or the inability to take measurements because the child was disabled or very thin (n=3). We further dropped observations with a phase angle >6.5 (n=13). Of the 356 households with migrant fathers, 309 recalled the sum of remittances and were included in the RQ 5 analysis.

Mean HAZ dropped steadily in the first 2 years of life but increased again by 6 years (table 1). Only 32% of fathers had been abroad before the child was born, but the fraction of fathers who had migrated for work in the previous time period increased steadily as the child aged (table 1). At the last measurement, 65% of the fathers had worked overseas at some point between the child’s second and sixth birthday. One third (33%, n=173) of fathers had never migrated for work within the recall period. Children had low lean and fat mass z-scores at 6 years (table 2).

**Table 1:** Sample characteristics

	Birth	6 months	1 year	2 years	6 years
	% (*n*)	% (*n*)	% (*n*)	% (*n*)	% (*n*)
Total N	524	513	505	500	524
Father’s migration in the previous time period					
No migration	67.9% (356)	65.3% (335)	59.2% (299)	51.2% (256)	35.3% (185)
1–12 months	13.4% (70)	15.8% (81)	14.9% (75)	12.2% (61)	6.5% (34)
>12 months	18.7% (98)	18.9% (97)	25.9% (131)	36.6% (183)	58.2% (305)
Other household member(s) worked abroad in the previous time period	9.2% (48)	9.6% (49)	10.9% (55)	13.0% (65)	22.5% (118)
Total costs of father’s migration in 100k NPR*, median (IQR)					2.0 (1.6–3.1)
Total amount of father’s remittances in 100k NPR*, median (IQR)					6.0 (2.8–11.1)
Net remittances in 100k NPR*, median (IQR)					3.2 (0.7–8.6)
Maternal height (cm), mean (SD)					150.9 (5.5)
Mother’s education					
Never went to school	71.7% (374)				
Primary to lower secondary (class 1–8)	17.1% (89)				
Secondary and above (class≥9)	11.3% (59)				
Father’s education					
Never went to school	44.8% (235)				
Primary to lower secondary (class 1–8)	32.2% (169)				
Secondary and above (class≥9)	23.0% (121)				
Household suffers from any degree of food insecurity	29.6% (155)				26.1% (137)
Height-for-age *z*-score, mean (SD)	−1.0 (1.0)	−1.4 (1.0)	−1.7 (0.9)	−2.0 (1.0)	−1.6 (0.9)
Stunting	16.4% (86)	21.8% (112)	35.6% (180)	50.8% (254)	31.9% (167)
Weight-for-height *z*-score, mean (SD)	−1.1 (1.1)	−0.6 (1.0)	−1.4 (1.0)	−1.1 (1.0)	−1.3 (0.9)
Wasting	20.4% (100)	9.4% (48)	25.9% (131)	20.4% (102)	21.0% (110)
Weight-for-age *z*-score, mean (SD)	−1.4 (0.9)	−1.4 (1.0)	−1.9 (1.0)	−1.9 (1.0)	−2.0 (0.9)
Underweight	23.7% (123)	26.5% (136)	44.4% (224)	44.4% (222)	47.3% (248)
Child age (months), mean (SD)	0.1 (0.0)	6.5 (0.3)	12.1 (0.3)	22.7 (0.5)	73.6 (1.9)
Child is a girl	47.7% (250)	48.1% (247)	47.7% (241)	47.6% (238)	47.7% (250)

* These variables have diverging numbers of observations: total costs of father’s migration *n*=345, total amount of father’s remittances *n*=319, net remittances *n*=309.

NPR, Nepalese Rupee.

**Table 2:** Specific growth and body composition outcomes at 6 years (*n*=525)

	Mean (SD)	*n*
Outcomes at age 6 years		
Body circumferences (cm)		
Head	48.2 (1.3)	524
Mid-upper arm (MUAC), median (IQR)	15.1 (14.4–16.1)	525
Waist, median (IQR)	49.2 (47.5–51.0)	525
Hip, median (IQR)	52.5 (50.5–54.2)	525
Calf	20.3 (1.4)	525
Skinfold thickness (mm)		
Biceps, median (IQR)	4.1 (3.7–4.8)	524
Triceps, median (IQR)	6.0 (5.1–7.1)	523
Subscapular, median (IQR)	4.6 (4.1–5.1)	521
Suprailiac, median (IQR)	5.2 (4.4–6.2)	524
Body composition		
Lean mass (kg)	14.0 (1.7)	472
Lean mass index (kg/m^2^)	11.8 (0.9)	472
Lean mass z-score*	−1.9 (0.9)	472
Fat mass (kg), median (IQR)	2.2 (1.5–2.8)	472
Fat mass index (kg/m^2^), median (IQR)	1.8 (1.3–2.4)	472
Fat mass z-score*, median (IQR)	−1.6 (-2.4–-0.9)	472
Other outcomes		
Tibia length (cm), median (IQR)	235.5 (226.5–245.5)	515
Grip strength (kg), median (IQR)	6.6 (5.9–7.6)	490
Grip strength		524
<5 kg	6.4% (34)	
5–<6.5 kg	41.0% (217)	
6.5–<8 kg	35.2% (186)	
≥8 kg	17.2% (91)	
Total *n*		525

* Calculated using UK reference data (Wells *et al*).^15^

### Regression results

#### Overall association between fathers’ migration and child growth (RQ 1)

**Figure 2:**
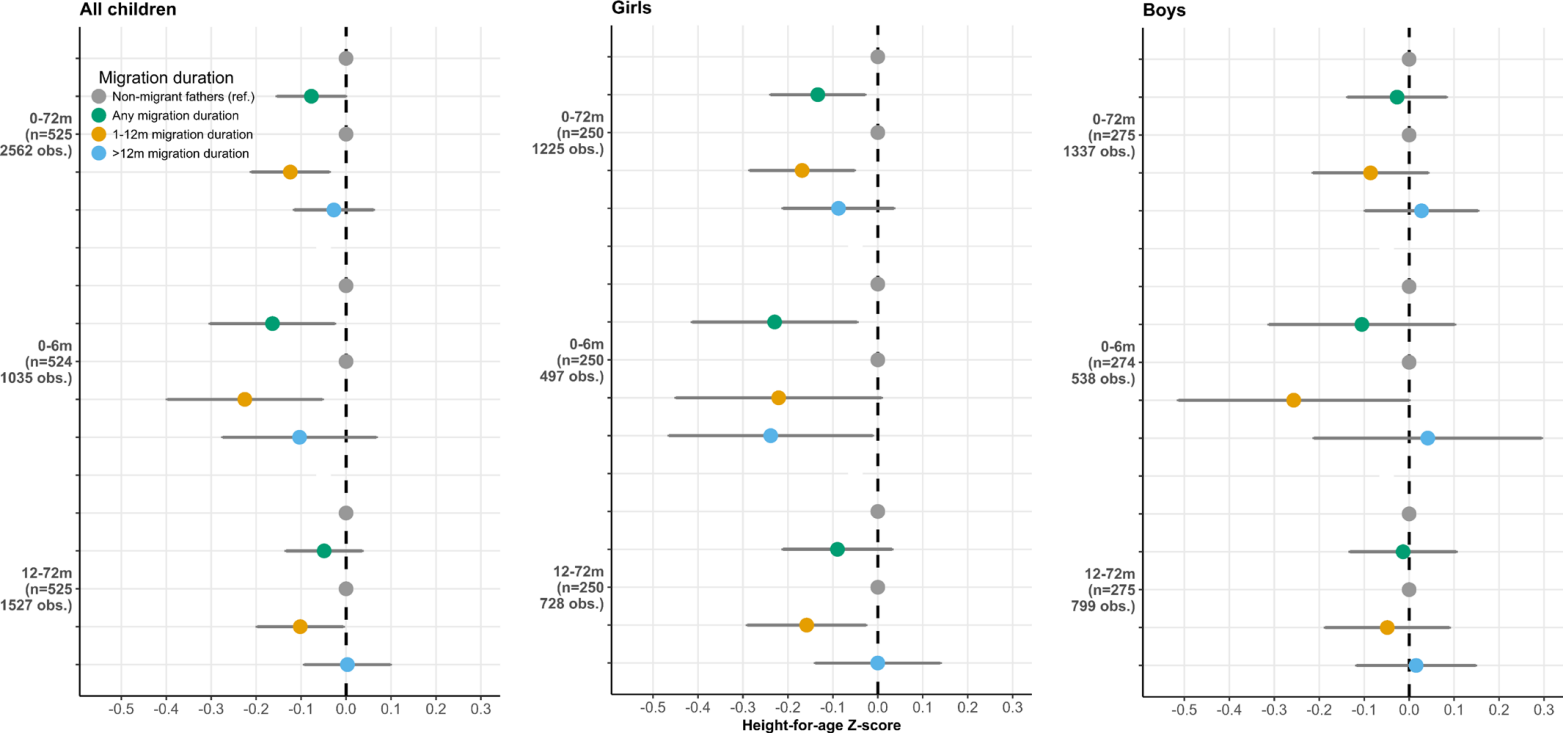
Regression estimates on the association between father’s migration and the duration of his stay abroad on child height-for-age *z*-score, at 0–72 months, 0–6 months and 12–72 months (complete sample, and stratified by child gender). Results from mixed-effects linear regression models.

Compared with children whose father had not worked overseas in the previous time period, children of labour migrants had −0.08 (95% CI −0.15 to –0.00) lower HAZ (figure 2 and online supplemental table 4.1). Unadjusted results are similar and presented in online supplemental table 4.2. Stratifying the sample by child gender showed that the negative association was only apparent in girls (−0.13, 95% CI −0.24 to –0.03) but not boys (−0.03, 95% CI −0.14 to 0.08) (figure 2 and online supplemental tables 4.3.1 and 4.3.2). The adjusted GVIFs were all <1.1, indicating negligible multicollinearity.

#### Differences in the association between father’s migration and child growth by duration of absence (RQ 2)

Children of migrants who had been abroad ≤12 months had −0.12 (95% CI −0.21 to –0.04) lower HAZ compared with children of non-migrants. Children of longer-term migrants (>12 months) showed no difference compared with children of non-migrants (figure 2 and online supplemental table 4.1). Unadjusted results are similar and presented in online supplemental table 4.2. Again, the observed association was only apparent in girls (−0.17, 95% CI −0.28 to –0.05) but not boys (−0.09, 95% CI −0.21 to 0.04).

#### Differences in the association between father’s migration and child growth by child age (RQ 3)

Separating the dataset by child age (0–6 months vs 12–72 months) showed that the association of HAZ with the fathers’ migration was only apparent in the younger children (−0.16, 95% CI −0.30 to –0.01), but not the older ones (−0.05, 95% CI −0.14 to 0.03). Stratifying by child gender showed that the negative association in young infants was only apparent in girls (−0.23, 95% CI −0.41 to –0.05), but not boys (−0.11, 95% CI −0.31 to 0.1). Further differentiating by the duration of the father’s migration showed that in both age-periods only the growth of children of recent migrants (≤12 months) was negatively affected (age-period 0–6 months: −0.23, 95% CI −0.40 to –0.05; 7–72 months: −0.10, 95% CI −0.20 to –0.01) (figure 2 and online supplemental table 4.1). However, stratifying by child gender revealed differing patterns: in female infants, we observed a negative association even after the father had been away for >12 months (−0.24, 95% CI −0.46 to –0.01), in boys the negative association was only apparent in infancy and when the father recently migrated (−0.26, 95% CI −0.51 to 0). Although the lack of precision in the estimates does not allow inferences about differences between earlier and later age-periods, there appears to be a pattern in that the estimates for children between birth and 6 months are consistently lower than for children aged 12–72 months, indicating that younger children may be more negatively affected by the fathers’ migration. Unadjusted results are similar and presented in online supplemental table 4.2.

#### The association between father’s migration and other measures of child growth at six years (RQ 4)

**Figure 3:**
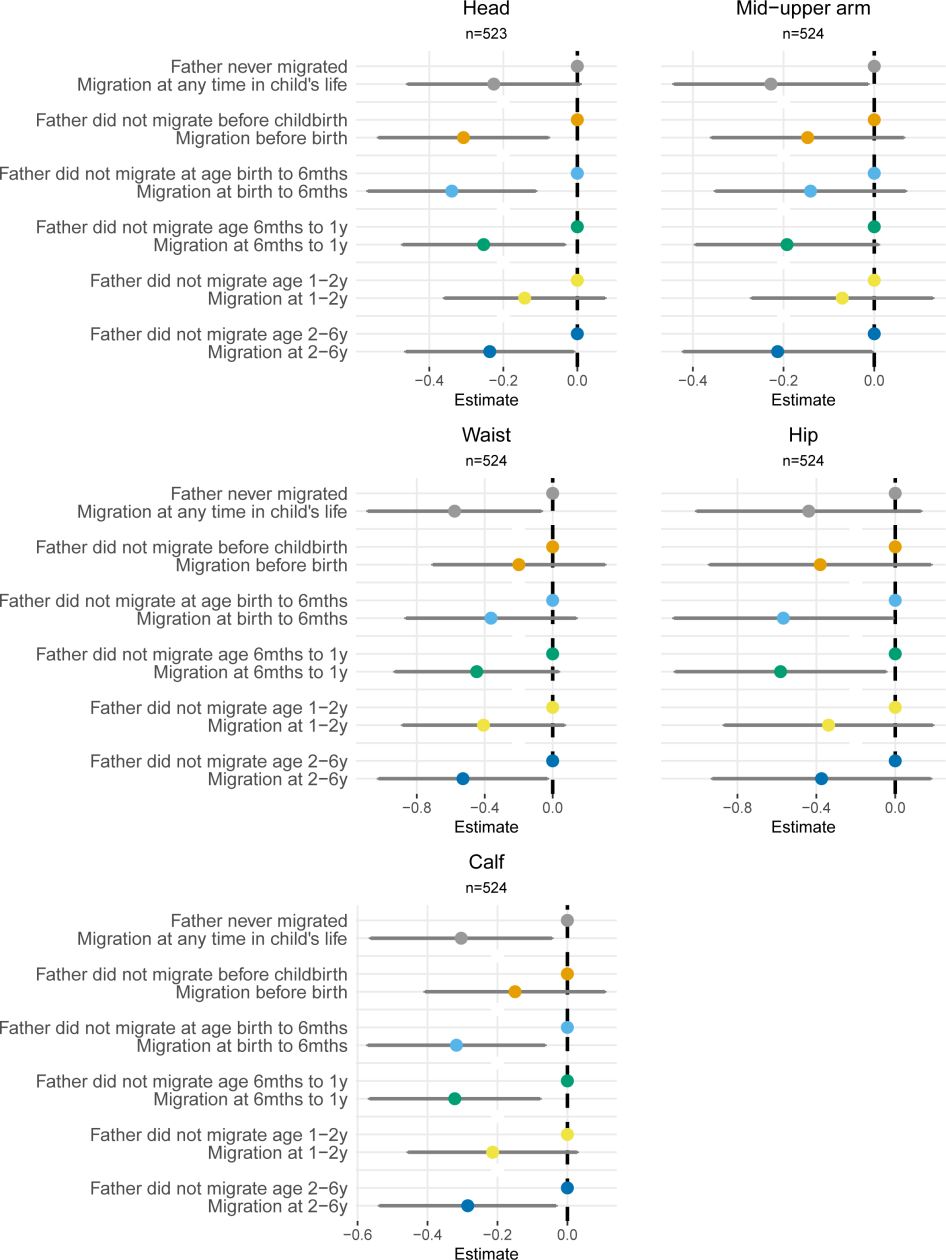
Association between father’s migration and child body circumferences (in cm) at 6 years of age.

Children whose fathers migrated at any time in the 7-year recall period had smaller waist and calf circumference (figure 3), smaller subscapular and supra-iliac skinfold thickness and lower lean mass than children of non-migrant fathers (online supplemental figures 5.1 and 5.2). Differentiating by the timing of migration relative to the child’s age showed that migration in some age-periods was associated with smaller body sizes (figure 3 and online supplemental figures 5.1 and 5.2). Children whose father migrated before childbirth or during infancy had smaller head circumferences (figure 3). While many estimates did not reach or only just reached significance and CIs were generally wide, it should be noted that there was a general pattern towards slightly smaller body sizes in left-behind children compared with children of non-migrants.

We found no association between father’s labour migration and tibia length or grip strength (online supplemental figure 5.3).

#### The impact of father’s remittances on child HAZ and other measures of growth at 6 years (RQ 5)

We found no association between cumulative net remittances sent by the father over the 7-year recall period and any measure of child growth at 6 years (online supplemental figure 6).

## Discussion

### Summary and interpretation of findings

#### Overall association between fathers’ migration and child growth (RQ 1)

Counter to our hypothesis of a positive effect of migration on child growth, we found that children of migrant fathers are shorter for their age than children of non-migrant fathers. This aligns with evidence from other studies and contexts.^22^

#### Differing associations by duration of migration (RQ 2)

Only children of fathers who had recently left were negatively affected. There was no difference in terms of HAZ between children of longer-term migrants and non-migrants. This aligned with our hypothesis and may be explained by the disruptive effects of migration on left-behind families.^23^,^26^ To our knowledge, no other study has investigated the effects of recent migration on child growth, but studies from Mexico found disruptive effects of migration on left-behind families. Kanaiaupuni and Donato^23^ found that each additional trip to the USA was associated with lower odds of infant survival in the left-behind family, whereas each additional month of experience in the USA was associated with improved odds of infant survival. Hamilton *et al*^27^ also found increased odds of infant mortality associated with recent migration of a household member to the USA. In Mexico, wives of recent international migrants, who received remittances for less than 1 year, had higher odds of having a low-birthweight baby while no association was found in women who received remittances for more than 1 year.^25^

#### Differing associations by child age (RQ 3)

In contrast to our initial hypothesis**,** after separating the sample by age group, we found a negative association between father’s migration and child HAZ at ages 0–6 months, but no difference between children of migrants and non-migrants aged 12–72 months. This is consistent with evidence from China where boys left behind in early childhood (age <6 years) were shorter for their age than sons of non-migrants.^28^ No differences were found in girls or between children of labour migrants and non-migrants at later ages.^28^ In Mexico, Lu^29^ found that parental international migration had a stronger negative association with HAZ in younger left-behind children, but found no such pattern in Indonesia. A study from Guatemala found a larger *positive* association between HAZ and living in a household with any migrant household member in children under 30 months compared with children aged 30 to 71 months.^30^

Growth velocity is the highest in the first few months of life,^31 32^ so insults to healthy development at this time have a particularly detrimental effect. However, the pathways through which the father’s migration affects the baby’s growth are not clear. One potential pathway is through elevated levels of distress in the mother. In the same area of Nepal as our study, women described their husband’s absence as a source of distress because he could not provide support, especially after childbirth or during illness (Clarke *et al*^33^). A hospital-based study in Lumbini, Nepal, compared reproductive health, depression and experiences of gender-based violence among wives of international labour migrants and non-migrants. Left-behind women had higher depression scores and were more likely to experience gender-based violence by in-laws and neighbours.^34^ Distressed mothers are more likely to experience breastfeeding problems,^35^ and stress impairs the breast milk supply.^36^ Meanwhile, reducing stress improves infant milk intake^37^ and weight gain.^38^ A second pathway is the lack of support from the migrant husband during pregnancy and in the postpartum period. Aryal *et al*^34^ found that left-behind women attended fewer antenatal care visits, were less likely to report adequate diet, rest and support during pregnancy or postpartum and were more likely to experience postpartum complications.

#### Differences in child gender

We observed differing patterns between girls and boys. In general, the negative associations were stronger and sometimes only apparent in girls. One exception is father’s recent migration in early infancy where the effect estimate was slightly larger for boys than girls. However, lack of precision in the estimates cautions against overinterpreting these results. On the one hand, these gender differences were surprising because in an earlier analysis on the determinants of infant growth in this cohort, we found no differences between boys and girls across any of the determinants studied.^12^ On the other hand, in the light of the prevailing gender norms and the roles of women and girls in this society, the results are plausible. In Dhanusha, as in many areas of Nepal and South Asia, women and girls are disadvantaged by traditional norms and practices such as patrilocal residence patterns, dowry system and early marriage, to name a few. It is therefore conceivable that the girls’ lower social status may lead to differences in caregiving during infancy. The interaction between gendered caregiving and fathers’ migration could not be addressed in this analysis and remains to be addressed in future studies.

#### Associations between fathers’ migration and other measures of child growth (RQ 4)

Only some outcomes showed significant results for exposure to migration and only in certain age-periods in the child’s life. It is difficult to ascertain the reasons for these differences between outcomes and exposure periods, and we want to refrain from overinterpreting these results. Nevertheless, a consistent pattern emerged showing that left-behind children’s skinfold thicknesses, body circumferences and lean mass tended to be smaller than their peers whose fathers did not migrate. This is consistent with results of the main analysis on HAZ, which showed that left-behind children did not grow as well as those of non-migrant fathers. Fat mass and lean mass showed differing patterns. Compared with children who had never migrated, left-behind children had lower lean mass, but not fat mass. This is consistent with Wells^39^ who found lower lean mass, but not fat mass, in children who had experienced undernutrition. This may be because the body preserves fat stores, which may offer short-term benefits for survival, at the expense of functional tissue.^39^ Another noteworthy finding is that children whose father migrated while they were very young had smaller head circumference at 6 years than children whose father was not absent in these time periods. Head circumference is most sensitive to malnutrition in early life, which is consistent with our findings where differences were only apparent in children whose fathers migrated during their first months of life. The observed associations, although small, could hint at lasting implications for the children’s brain development and later schooling.^40 41^

#### Associations between fathers’ remittances and child growth (RQ 5)

Contrary to our hypothesis, we found no association between remittances and attained HAZ or other outcomes at 6 years. This aligns with studies in other populations.^2442^,^44^

Plausible explanations for the lack of an effect include first that most remittance income is used for purposes that do not impact child growth and/or well-being. Of the migrant households included in this study, many reported using remittances to pay for everyday consumption other than food, paying off the migration loan or other loans that they had, to build or rebuild their house or to finance special occasions such as wedding or funeral.^11^ These expenses make up large shares of the overall remittances so that a relatively small amount may be left for spending that benefits the child. Second, there is a time lag between the migrants’ departure and their first remittances, and early remittances may be used to pay off migration loans. Hence, it takes time until remittances are available for spending that benefits left-behind children, and funds are unlikely to be available shortly after departure when children seem to be most vulnerable. Unfortunately, we lack data on changes in remittance spending over time to corroborate this.

Another analysis on this cohort^12^ found that low birth weight, as a measure of intrauterine growth restriction, was the most important determinant of infant growth. Plausibly, use of remittance income could improve women’s nutritional status before and during pregnancy, and also growth of the baby. However, research in the same area of Nepal found that intra-household food allocation is inequitable and that pregnant women get smaller shares of food and nutrients compared with other adult household members.^45^ This might explain the lack of association between remittances and child growth.

### Study strengths and limitations

Our analysis offers new insights into the effects of labour migration on left-behind children. There is a dearth of evidence on *international* migration including from major remittance-dependent countries, like Nepal. Unlike other studies of associations between labour migration and left-behind children that rely on secondary data collected for other purposes,^2429 46^,^51^ we collected the data specifically for this purpose. This provided quality-controlled and detailed information regarding the migration patterns enabling accurately linking of migration cycles to periods in the child’s life. Using our longitudinal dataset reduces the risk of reverse causation inherent in cross-sectional studies. A systematic review found only four of 29 studies (14%) were based on longitudinal data and those only had two data points per child.^22^ The detailed migration information and longitudinal growth data enabled us to investigate potential moderating factors such as child age, duration of migration and remittance income, and identify periods in the child’s life when they may be more sensitive to the disrupting effects of the father’s migration. Apart from the 6-year measurement point, this study focuses on growth during the first 1000 days when children are most susceptible to insults limiting their growth. With the exception of Davis and Brazil,^24^ evidence outside of China draws on data from older children.^29 49 52 53^

Our study has some limitations. As with all observational research, we must be cautious about claims of causality. We visualised potential causal mechanisms and chose covariates to adjust for in our regression analysis using a DAG. However, a possibility of residual confounding remains if a covariate did not appropriately signal the respective factor, for example, if years of schooling did not appropriately capture the fathers’ education level and job qualification.

Data limitations prevented us from addressing some potentially important factors in our analysis. Household size and composition may be confounders as they may be associated with both the decision to seek work abroad and the nutritional status of children in the home. We argue that maternal mental health and stress are likely mediating or moderating factors in the association between paternal labour migration and child growth, but we were unable to test this mechanism in our analysis due to a lack of data.

Our sample size was small compared with other studies on this topic that used existing secondary datasets.^24 29 49^ But among those studies that, like ours, analysed primary data, sample sizes were comparable,^5254^,^57^ with the exception of one larger Chinese study.^58^

The analysis of the association between migration at different time points in the child’s life and measures of child growth other than height (RQ 4) must be interpreted with caution. The measures of exposure to migration differentiate poorly between children left behind and children whose fathers did not migrate, since children who were categorised as left behind at one point may have been categorised as children of non-migrants at all other points, and vice versa. For example, about half of the fathers who were categorised as non-migrants between birth and 6 months migrated later in their child’s life, and this may have impacted the growth outcomes at 6 years. These indicative findings, in the context of a nuanced longitudinal analysis of the outcome HAZ, paint a coherent picture of poorer growth in left-behind children compared with children of non-migrants.

Data on remittances relied on respondents’ recall with no means to verify accuracy, and financial questions are sensitive, so there is a possibility that respondents did not correctly indicate the amount of remittances a household received from the respective migrant. Respondents may have wanted to present themselves as poorer or more economically successful than they actually were, or they did not accurately remember. The extent of potential bias in the reported remittance data could not be assessed.

The findings of this study may have limited generalisability outside of Nepal or even outside of Dhanusha district. The labour migration patterns found in labour-sending countries vary in important aspects, and so the implications for the left-behind families and in particular the children will be different.

### Implications for research, programming and policy

These findings and limitations point towards areas for future work. Qualitative research could provide insights into the experiences of pregnant women and mothers of young babies whose partner is preparing for or who has already left for overseas employment. Knowledge about these women’s living situation, needs, stressors and coping strategies could inform interventions aimed at supporting them in the transition phase in the months around the husband’s departure. It could also help gain a better understanding of attitudes towards and caring practices for daughters.

Existing programmes working towards improving outcomes of labour migration could incorporate some of the insights gained in this study to better cater their work towards the needs of (prospective) migrants and their families. We found that pregnant women and those with young babies are vulnerable and need targeted support. Additionally, programmes providing migration counselling and support could inform new or expecting fathers about their family’s vulnerabilities and advise them on how best to support them.

Some of the negative associations observed may be partly explained by the financial risks and overall uncertainties surrounding migration. To alleviate some of the stresses and generally increase the benefits of migration for migrants and their families, the Government of Nepal must commit to implementing existing policies designed to make migration safer and more financially rewarding for migrants.

## Conclusion

We found no evidence of a positive association between paternal labour migration and the growth of the left-behind children. Instead, at a very young age (≤6 months) and during the disruptive period within 12 months of when the father went abroad, left-behind children grew less well than children whose fathers did not migrate. We also found that girls are more strongly affected than boys. It remains unclear whether these insults to their growth are permanent or can be compensated for through longer growth periods and later puberty. Targeted support is needed for pregnant and recently delivered wives of migrants to improve their diets, child feeding practices, care seeking and gendered childcare practices. Labour migration policies may need to be adjusted to better serve migrants and their families.

## Supplementary material

10.1136/bmjgh-2025-021253 online supplemental file 1

## Data Availability

Data are available in a public, open access repository.

## References

[1] IOM (2024). World migration report 2024. McAuliffe M, Oucho LA, eds.

[2] National Planning Commission (Nepal) (2020). Nepal multiple indicator cluster survey 2019.

[3] World Bank Group (2025). Personal remittances, received (% of GDP) 2023. http://data.worldbank.org/indicator/BX.TRF.PWKR.DT.GD.ZS?view=map&year_high_desc=true.

[4] Wu J, Kilby P, Mathema J (2023). The Precarity of Women’s Short-Term Migration: A Case Study from Nepal. Migr Dev.

[5] Amnesty International (2011). False promises: exploitation and forced labour of Nepalese migrant workers.

[6] Republica (2024). Over 4,000 migrant Nepali workers died abroad in three years.

[7] Regmi P, Aryal N, Bhattarai S (2024). Exploring lifestyles, work environment and health care experience of Nepalese returnee labour migrants diagnosed with kidney-related problems. PLoS One.

[8] Wasti SP, Babatunde E, Bhatta S (2024). Nepali Migrant Workers and Their Occupational Health Hazards in the Workplace: A Scoping Review. Sustainability.

[9] Tanner JM (1992). Growth as a measure of the nutritional and hygienic status of a population. Horm Res.

[10] de Onis M, Branca F (2016). Childhood stunting: a global perspective. Matern Child Nutr.

[11] Busert-Sebela L (2023). The association between paternal labour migration and the growth of the left-behind children in the plains of Nepal.

[12] Busert‐Sebela L, Cortina‐Borja M, Paudel V (2025). Determinants of Infant Growth in a Birth Cohort in the Nepal Plains. Matern Child Nutr.

[13] WHO Multicentre Growth Reference Group (2006). WHO child growth standards: length/height-for-age, weight-for-age, weight-for-length, weight-for-height and body mass index-for-age. Methods and development.

[14] de Onis M, Onyango AW, Borghi E (2007). Development of a WHO growth reference for school-aged children and adolescents. Bull World Health Organ.

[15] Wells JCK, Williams JE, Chomtho S (2012). Body-composition reference data for simple and reference techniques and a 4-component model: a new UK reference child. Am J Clin Nutr.

[16] Greenland S, Pearl J, Robins JM (1999). Causal Diagrams for Epidemiologic Research. Epidemiology (Sunnyvale).

[17] Textor J, Hardt J, Knüppel S (2011). DAGitty: a graphical tool for analyzing causal diagrams. Epidemiology (Sunnyvale).

[18] Coates J, Swindale A, Bilinsky P (2007). Household food insecurity access scale (HFIAS) for measurement of food access: indicator guide (v. 3).

[19] Pinheiro J, Bates D, R Core Team (2025). nlme: Linear and nonlinear mixed effects models.

[20] Fox J, Weisberg S (2019). An R companion to applied regression.

[21] Dataset] Busert-Sebela L, Cortina-Borja M, Eaton S (2026).

[22] Fellmeth G, Rose-Clarke K, Zhao C (2018). Health impacts of parental migration on left-behind children and adolescents: a systematic review and meta-analysis. Lancet.

[23] Kanaiaupuni SM, Donato KM (1999). Migradollars and mortality: the effects of migration on infant survival in Mexico. Demography.

[24] Davis J, Brazil N (2016). Migration, Remittances and Nutrition Outcomes of Left-Behind Children: A National-Level Quantitative Assessment of Guatemala. PLoS One.

[25] Frank R (2005). International migration and infant health in Mexico. J Immigr Health.

[26] Antman FM, Constant AF, Zimmermann KF (2013). International handbook on the economics of migration.

[27] Hamilton ER, Villarreal A, Hummer RA (2009). Individual, Household, and Community U.S. Migration Experience and Infant Mortality in Rural and Urban Mexico. Popul Res Policy Rev.

[28] Zhang N, Bécares L, Chandola T (2015). Does the timing of parental migration matter for child growth? A life course study on left-behind children in rural China. BMC Public Health.

[29] Lu Y (2015). Internal migration, international migration, and physical growth of left-behind children: A study of two settings. Health Place.

[30] Carletto C, Covarrubias K, Maluccio JA (2011). Migration and child growth in rural Guatemala. Food Policy.

[31] Lejarrage H, Cameron N, Bogin B (2012). Human growth and development.

[32] WHO (2009). The WHO child growth standards: growth velocity based on weight, length and head circumference: methods and development.

[33] Clarke K, Saville N, Bhandari B (2014). Understanding psychological distress among mothers in rural Nepal: a qualitative grounded theory exploration. BMC Psychiatry.

[34] Aryal S, Shrestha D, Pant SB (2019). Reproductive Health Issues and Depression in Wives of Labor Migrant Workers. J Nepal Health Res Counc.

[35] Dennis CL, McQueen K (2007). Does maternal postpartum depressive symptomatology influence infant feeding outcomes?. Acta Paediatr.

[36] Dewey KG (2001). Maternal and fetal stress are associated with impaired lactogenesis in humans. J Nutr.

[37] Mohd Shukri NH, Wells J, Eaton S (2019). Randomized controlled trial investigating the effects of a breastfeeding relaxation intervention on maternal psychological state, breast milk outcomes, and infant behavior and growth. Am J Clin Nutr.

[38] Dib S, Wells JCK, Eaton S (2022). A Breastfeeding Relaxation Intervention Promotes Growth in Late Preterm and Early Term Infants: Results from a Randomized Controlled Trial. Nutrients.

[39] Wells JCK (2019). Body composition of children with moderate and severe undernutrition and after treatment: a narrative review. BMC Med.

[40] Winick M, Rosso P (1969). Head circumference and cellular growth of the brain in normal and marasmic children. J Pediatr.

[41] Tiwari K, Goyal S, Malvia S (2017). Impact of malnutrition on head size and development quotient. *Int J Res Med Sci*.

[42] Kroeger A, Anderson KH (2014). Remittances and the human capital of children: New evidence from Kyrgyzstan during revolution and financial crisis, 2005–2009. J Comp Econ.

[43] Antén J-I (2010). The Impact of Remittances on Nutritional Status of Children in Ecuador. International Migration Review.

[44] Ponce J, Olivié I, Onofa M (2011). The role of international remittances in health outcomes in Ecuador: prevention and response to shocks. Int Migr Rev.

[45] Harris-Fry HA, Paudel P, Shrestha N (2018). Status and determinants of intra-household food allocation in rural Nepal. Eur J Clin Nutr.

[46] Ban L, Guo S, Scherpbier RW (2017). Child feeding and stunting prevalence in left-behind children: a descriptive analysis of data from a central and western Chinese population. Int J Public Health.

[47] Chen C, He W, Wang Y (2011). Nutritional status of children during and post-global economic crisis in China. Biomed Environ Sci.

[48] Mu R, de Brauw A (2015). Migration and young child nutrition: evidence from rural China. J Popul Econ.

[49] Schmeer KK (2013). Family structure and child anemia in Mexico. Soc Sci Med.

[50] Kunwar R, Vajdic CM, Muscatello DJ (2020). Parental international migration is not associated with improved health care seeking for common childhood illnesses and nutritional status of young children left-behind in Nepal. Public Health (Fairfax).

[51] Islam MM, Khan MN, Mondal MNI (2019). Does parental migration have any impact on nutritional disorders among left-behind children in Bangladesh?. Public Health Nutr.

[52] Graham E, Jordan LP (2013). Does Having a Migrant Parent Reduce the Risk of Undernutrition for Children Who Stay Behind in South-East Asia?. Asian Pac Migr J.

[53] Viet Nguyen C (2016). Does parental migration really benefit left-behind children? Comparative evidence from Ethiopia, India, Peru and Vietnam. Soc Sci Med.

[54] Mo X, Xu L, Luo H (2016). Do different parenting patterns impact the health and physical growth of “left-behind” preschool-aged children? A cross-sectional study in rural China. Eur J Public Health.

[55] Onyango A, Tucker K, Eisemon T (1994). Household headship and child nutrition: a case study in western Kenya. Soc Sci Med.

[56] Wickramage K, Siriwardhana C, Vidanapathirana P (2015). Risk of mental health and nutritional problems for left-behind children of international labor migrants. BMC Psychiatry.

[57] Tao S, Yu L, Gao W (2016). Food preferences, personality and parental rearing styles: analysis of factors influencing health of left-behind children. Qual Life Res.

[58] Gao Y, Li LP, Kim JH (2010). The impact of parental migration on health status and health behaviours among left behind adolescent school children in China. BMC Public Health.

