# Supplementary material for: The association between paternal labour migration and the growth of the left-behind children—evidence from a birth cohort in Dhanusha district, Nepal
Source: BMJ Glob Health. 2026 Jan 9;11(1):e021253. doi: 10.1136/bmjgh-2025-021253 (PMC12815186; doi:10.1136/bmjgh-2025-021253)
Supplement: online supplemental file 1 [file bmjgh-11-1-s001.docx]

# Supplementary material

For the paper titled “The association between paternal labour migration and the growth of the left-behind children - evidence from a birth cohort in Dhanusha district, Nepal” (Busert et al.)

# Isotope calibration sub-study

**Background and aims**

Almost all countries, including Nepal (Ministry of Health and Population [Nepal], New ERA, & Inc., 2017), have simultaneously experienced an increase in the prevalence of overweight and obesity, which is a risk factor for many non-communicable diseases such as type 2 diabetes and cardiovascular disease (Swinburn et al., 2011). A double burden of overweight and stunting in children has been observed in many Low and Middle Income Countries (LMICs) (Fernald & Neufeld, 2007; Keino, Plasqui, Ettyang, & Van Den Borne, 2014; Popkin, Richards, & Montiero, 1996) and overweight children tend to grow into overweight adults (Singh, Mulder, Twisk, Van Mechelen, & Chinapaw, 2008). Most research studying the risks factors and consequences of overweight and obesity uses body mass index (BMI, body weight/height^2^) as the indicator of body fatness, as both constituents are relatively easy to measure. Since BMI correlates not only with fat mass but also lean mass, and a given BMI can comprise a wide range of body fat (Wells, 2000), it is a poor indicator of fatness.

The most frequently used model of body composition is the two-component model which divides the body into fat mass (FM) and lean mass (LM). Body water makes up the largest proportion of LM, but the level of hydration depends on the individual’s age (Wells et al., 2010). With estimates of total body water (TBW) and hydration, LM can be calculated. FM is the difference between total body weight and LM.

Deuterium dilution is the gold-standard for estimating total body water, but it is time consuming, expensive, requires very specialised equipment and is therefore less suitable for population-based studies and research in low-resource settings. Bioelectrical impedance analysis (BIA) constitutes a portable, simple to use and non-invasive method of measuring body composition. The prediction of TBW from BIA measurements, however, requires specific prediction equations for the respective population due to differences in body geometry, specifically variability in limb segment girths, and differences in the ratios of segments to trunk. Most prediction equations built into BIA machines are derived from white European populations with poor validity in other ethnic populations (Haroun et al., 2010). The main aim of this sub-study was to calibrate the Bodystat 500 Touch by estimating total body water using deuterium oxide in a sub-sample of children in the GMS cohort.

**Selection of participants**

The isotope calibration study was conducted over one month after the main data collection had been completed. We aimed to sample 60 children (30 girls), equally distributed over the range of weights observed in our population (10.9 - 26.0kg) (Devakumar et al., 2015). Children were purposively selected from the main study cohort and were all six years old.

**Preparation of doses**

Deuterium doses and other equipment were prepared in the office in Janakpur. We used 0.07g of stock deuterium oxide (~ 99.9%) per kg of the child’s body weight, which was obtained from the records of the main data collection. For example: 1.19g deuterium oxide for a child weighing 17 kg. Fifty grams (50g) of water was added to the deuterium. The dosing bottle and a straw were put into a resealable plastic bag and weighed using a Sartorius TE212 (Sartorius AG, Germany) accurate to 0.01g. The cotton buds in the salivettes were cut in half because the children found it difficult to handle the relatively large piece of cotton as a whole. The dosing bottle and salivettes were pre-labelled with the child’s ID number.


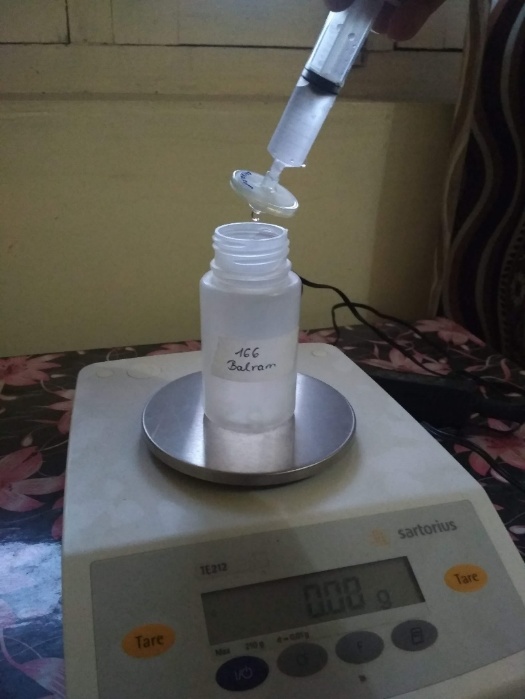

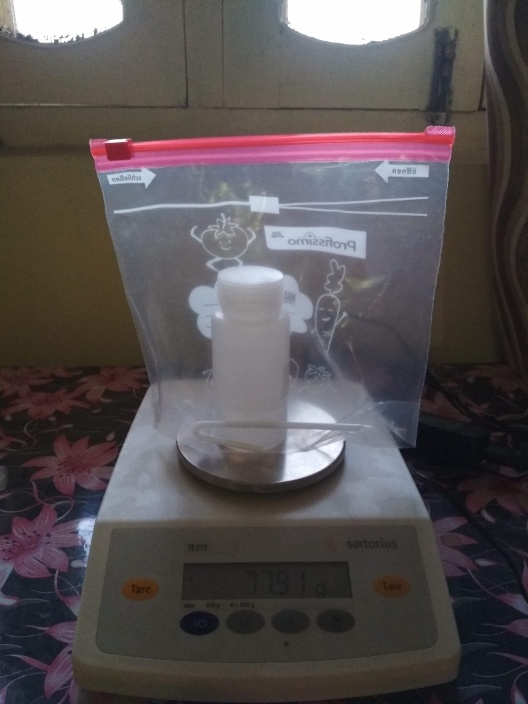


**Supplementary Figure 1.1: Preparation of deuterium doses.**

**Collection of samples**

The selected children were visited in their homes. The data collector (SSY) explained the procedures to the mother or guardian and took her/his consent. We asked the family when the child last ate or drank anything. If it had been less than half an hour, he waited before taking the first sample. To collect the saliva sample, the child was advised to first collect saliva in his/her mouth and then take half a cotton bud, roll it around in the mouth for one to two minutes until it was fully soaked in saliva. The soaked bud was then put back in the salivette and the procedure was repeated with the second half of the cotton bud.

The child was then asked to drink the deuterium dose through the straw while the bottle was kept in the bag to catch possible spillage. When the child was finished the straw was put into the bottle, the bottle closed, and the bag sealed. Back in the office the weight of the empty bag was recorded.

A pre-weighed one litre drinking bottle with water was handed to the family and they were instructed to only let the child drink from this bottle until our return four hours later. We also provided a small carton (200ml) of fruit juice as an incentive for the child and recorded whether s/he drank it.

The data collector returned to the household four hours later. Upon arrival he would ask the family when the child last ate or drank anything and would wait for up to half an hour before collecting the second saliva sample or taking the BIA measurements. He asked the family to return the drinking bottle and weighed it using the Tanita BD-590 Pediatric Scale accurate to 0.01kg to determine the amount of water that the child had drunk during the four-hour waiting period. To thank the child for his/her participation, we gifted a pair of sweatpants and a long-sleeved shirt of known weights and asked the child to change into these clothes. This allowed us to control possible inaccuracies arising from differences in the children’s clothes. The data collector then took measurements of the child’s weight, height and BIA. Lastly, a second saliva sample was taken following the same procedure as for the first one.

**Sample analysis**

Samples were stored in a fridge in the project office in Janakpur until completion of the data collection. In Kathmandu, before bringing them to London, the salivettes were spun in a centrifuge rented at a local laboratory and the resulting saliva samples stored in a freezer.

The samples were analysed at the UCL Great Ormond Street Institute of Child Health. Isotopic enrichment of the samples was estimated using continuous flow isotope ratio mass spectrometry (IRMS), using Delta XP instrumentation (Thermo Fisher Scientific). Each sample was pipetted into Exetainer tubes and an aluminium rod with 5% platinum was added. The tubes were sealed, filled with hydrogen and left to equilibrate. All samples were analysed in duplicate and measured against five reference standards.

**Total body water (TBW) and lean mass (LM) estimation**

Using the results from IRMS analysis I calculated total body water (TBW) as shown in Supplementary Equation 1.1 (Davies & Wells, 1994)

$$TBW (kg)= \frac{\frac{A*T}{a}\times\frac{\left( Ed-Et \right)}{\left( Es-Ep \right)}}{1000\times1.044} -W$$

**Supplementary Equation 1.1: Total body water (TBW)**

Where $A$ is the mass of the isotope drink (dose) given to the child, a is the amount of isotope drink diluted for analysis in the IRMS, $T$ is the amount of diluent water used to dilute a. Average Isotope enrichment $E$ is expressed in delta (*δ*) units. $Ed$ is the average dose enrichment, $Et$ is the average diluent water enrichment, $Es$ is the average post-dose enrichment, $Ep$ is the average pre-dose enrichment. The correction factor 1.044 accounts for hydrogen ion exchange and 1000 transforms from g to kg. $W$ is the amount of water (in kg) that the child drank in the four-hour equilibration period.

Lean mass ($LM$) consists mostly of water, but the exact hydration levels vary by sex, age and nutritional status. Since the BMI-specific hydration factors established by Gutiérrez-Marín et al. (2019) mostly differentiate BMI-levels at the higher end of the range and the children in my sample are mostly (very) thin or normal weight, I decided to use the hydration factors which only differentiate by age and sex as established by Wells et al. (2010) for children between 6 and 8 years. The hydration factors used were 0.761 for boys and 0.753 for girls. Supplementary Equation 1.2 shows the equation used to calculate$LM$.

$$LM= \frac{TBW}{hydration factor}$$

**Supplementary Equation 1.2: Lean mass (LM)**

**Data cleaning**

I excluded children with a phase angle > 6.5 or $TBW$< 5 litres as these values are likely implausible and due to measurement error. I additionally used boxplots and scatterplots to detect outlying values.

**Description of analytical sample**

Sixty-three children were enrolled in the study. One child had a learning disability and was unable to provide a sample or drink the deuterium dose, so we could not complete the data collection. Two children had a phase angle > 6.5 and three children had $TBW$< 5 litres. We additionally excluded three children with very unlikely body composition results (e.g., extremely low FM relative to body weight) that were likely due to contaminations in the isotope sample collection. Supplementary Table 1.1 shows characteristics of the 54 children included in the analysis.

**Supplementary Table 1.1: Description of sample in the isotope sub-study**

|  | **Boys** | **Girls** |
| --- | --- | --- |
|  | *n*=27 | *n*=27 |
| Age (months) (median, IQR) | 78.8 (78.0-79.3) | 79.0 (77.9-79.8) |
| Height (cm) (mean, SD) | 109.8 (6.5) | 107.5 (7.2) |
| Weight (kg) (median, IQR) | 15.9 (14.9-18.9) | 16.5 (14.3-17.3) |
| Height-for-age *z*-score (mean, SD) | -1.8 (1.3) | -2.0 (1.3) |
| Stunting (*n*, %) | 14 (52%) | 15 (56%) |
| Body mass-index (kg/m^2^) (median, IQR) | 13.8 (12.9-14.9) | 13.9 (13.1-14.7) |
| Total body water (l) (mean, SD) | 10.8 (1.9) | 10.0 (1.5) |
| Lean mass (kg) (mean, SD) | 14.2 (2.5) | 13.3 (2.1) |
| Lean mass index (kg/m^2^) (mean, SD) | 11.7 (1.3) | 11.5 (1.0) |
| Fat mass (kg) (median, IQR) | 2.6 (2.1-3.6) | 3.1 (2.2-4.0) |
| Fat mass index (kg/m^2^) (mean, SD) | 2.4 (1.0) | 2.5 (0.9) |

**Prediction equations for lean mass and total body water**

Prediction of total body water from BIA relies on the assumption that the body is a cylinder-shaped conductor and the conduction material is water. The volume can be calculated using Supplementary Equation 1.3 (Kushner, 1992)

$$V= \rho\times\frac{l^{2}}{Z}$$

**Supplementary Equation 1.3: Prediction of body volume from impedance**

where $V$ is the volume (cm^3^), ρ = specific resistivity (ohm-cm), $Z$ = impedance (ohm), $l$ is the length of the conductor.

The prediction equations for $LM$ and $TBW$ were generated by performing a linear regression with $LM$ or $TBW$ as the dependent variable and impedance index (height (cm)^2^/impedance) as the independent variable.

The resulting prediction equations were:

$LM$ = 2.730 + 0.788 m^2^/Z

Coefficient of determination ($R^{2}$) = 0.80, root-mean-square error ($RMSE$) = 1.04

$TBW$ = 2.014 + 0.600 m^2^/$Z$

$R^{2}$ = 0.80, $RMSE$ = 0.80


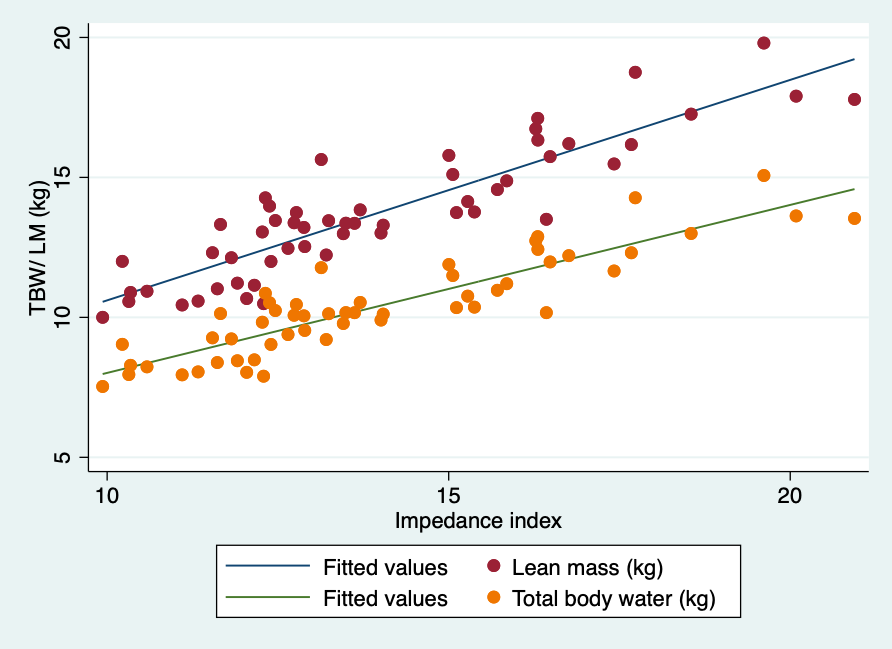


**Supplementary Figure 1.2: Scatter plot with regression line for lean mass and total body water from impedance index (height squared/impedance)**

**References**

Cleveland, W. S., Grosse, E., & Shyu, W. M. (1992). Local regression models. In J. M. Chambers & T. J. Hastie (Eds.), *Statistical Models in S*: Wadsworth & Brooks/Cole.

Davies, P. S., & Wells, J. C. (1994). Calculation of total body water in infancy. *Eur J Clin Nutr, 48*(0954-3007 (Print)), 490–495. Retrieved from <https://pubmed.ncbi.nlm.nih.gov/7956990/>

Devakumar, D., Grijalva-Eternod, C. S., Roberts, S., Chaube, S. S., Saville, N. M., Manandhar, D. S., . . . Wells, J. C. (2015). Body composition in Nepalese children using isotope dilution: the production of ethnic-specific calibration equations and an exploration of methodological issues. *PeerJ, 3*, e785. doi:10.7717/peerj.785

Fernald, L. C., & Neufeld, L. M. (2007). Overweight with concurrent stunting in very young children from rural Mexico: prevalence and associated factors. *Eur J Clin Nutr, 61*(5), 623-632. doi:10.1038/sj.ejcn.1602558

Gutiérrez-Marín, D., Luque, V., Ferré, N., Fewtrell, M. S., Williams, J. E., & Wells, J. C. K. (2019). Associations of age and body mass index with hydration and density of fat-free mass from 4 to 22 years. *Eur J Clin Nutr, 73*(10), 1422-1430. doi:10.1038/s41430-019-0447-4

Haroun, D., Taylor, S. J. C., Viner, R. M., Hayward, R. S., Darch, T. S., Eaton, S., . . . Wells, J. C. K. (2010). Validation of Bioelectrical Impedance Analysis in Adolescents Across Different Ethnic Groups. *Obesity, 18*(6), 1252-1259. doi:10.1038/oby.2009.344

Haug, S. (2008). Migration Networks and Migration Decision-Making. *Journal of Ethnic and Migration Studies, 34*(4), 585-605. doi:10.1080/13691830801961605

Keino, S., Plasqui, G., Ettyang, G., & Van Den Borne, B. (2014). Determinants of Stunting and Overweight among Young Children and Adolescents in Sub-Saharan Africa. *Food and Nutrition Bulletin, 35*(2), 167-178. doi:10.1177/156482651403500203

Kushner, R. F. (1992). Bioelectrical Impedance Analysis: A Review of Principles and Applications. *Journal of the American College of Nutrition, 11*(2), 199-209. doi:10.1080/07315724.1992.12098245

Ministry of Health and Population [Nepal], New ERA, & Inc., I. I. (2017). *Nepal Demographic and Health Survey 2016 - Full Report*. Retrieved from <https://www.dhsprogram.com/publications/publication-fr336-dhs-final-reports.cfm>

Popkin, B., Richards, M., & Montiero, C. (1996). Stunting is associated with overweight in children of four nations that are undergoing the nutrition transition. *Journal of Nutrition, 126*, 3009-3016.

Singh, A. S., Mulder, C., Twisk, J. W. R., Van Mechelen, W., & Chinapaw, M. J. M. (2008). Tracking of childhood overweight into adulthood: a systematic review of the literature. *Obesity Reviews, 9*(5), 474-488. doi:10.1111/j.1467-789x.2008.00475.x

Swinburn, B. A., Sacks, G., Hall, K. D., Mcpherson, K., Finegood, D. T., Moodie, M. L., & Gortmaker, S. L. (2011). The global obesity pandemic: shaped by global drivers and local environments. *The Lancet, 378*(9793), 804-814. doi:10.1016/s0140-6736(11)60813-1

Wells, J. C. K. (2000). A Hattori chart analysis of body mass index in infants and children. *International Journal of Obesity, 24*(3), 325-329. doi:10.1038/sj.ijo.0801132

Wells, J. C. K., Williams, J. E., Chomtho, S., Darch, T., Grijalva-Eternod, C., Kennedy, K., . . . Fewtrell, M. S. (2010). Pediatric reference data for lean tissue properties: density and hydration from age 5 to 20 y. *American Journal of Clinical Nutrition, 91*(3), 610-618. doi:10.3945/ajcn.2009.28428

# Calculation of exposures

## Calculation of father’s duration abroad in relation to the child’s lifetime

To determine the child’s exposure prior to the selected time points (i.e., birth, one year, two years, six years) we had to calculate the father’s exact dates of migration. In the 2018 follow-up questionnaire we had asked the respondents to only name the year and month that the migrant had left and returned. We did not ask for the day of the month because we suspected that most interviewees would not be able to remember and because that level of precision was not necessary. The dates were recorded in the Nepali calendar, but we had to convert them to the Gregorian calendar to be able to use the dates in statistical programmes. To do so, we assumed that the migrant had left/returned on the first day of the respective month in the Nepali calendar. After concatenating the event’s year, month and day into the format YYYYMMDD, we used the Stata command nepengdate (written by James Beard, not published) to convert them to the Gregorian calendar. We then subtracted the child’s date of birth from the date that the migrant had left or returned, which gave us the number of days that this event happened relative to the child’s date of birth. A negative number of days meant that the event had happened before the child was born, a positive number indicated the child’s age in days that the event had happened. For each of the selected time points (birth, six months, one year, two years, and six years) we calculated whether the child had been exposed to labour migration in the *preceding* time period, i.e., from the previous time point to this one (e.g., from birth to six months for time point 6 months, from six months to one year for time point one year, etc.).

If the child had been exposed to the father’s migration in the preceding time period, we recorded the duration of exposure to this particular cycle of migration. We counted all migration cycles that extended into the respective time period. If the respective cycle started in an earlier time period, then this cycle was counted as well in its full duration. If the child had been exposed to more than one cycle of migration within one period, the duration of all of these migration cycles were added up. This was mostly relevant in the period preceding 72 months (six years) because this period spanned four years (from two to six years) in which several migration cycles could have taken place. We also considered migration before conception, but only if the respective cycle of migration ended less than one year before childbirth.

Supplementary Figure 2.1, panel A visualises the paternal migration trajectories of three hypothetical examples in a timeline and demonstrates how these were summarised to convey the child’s exposure to migration. Child A’s father worked abroad for 24 months before the child was born and returned shortly before Child A’s birth. Therefore, the duration of exposure to migration in the period before birth is 24 months. In the growth periods from birth to six months and six months to one year the father was not abroad and child A is coded as not having been exposed to migration. In the period between 12 and 24 months, the father migrated and had been away for eight months by the time the child was 24 months old. This migration cycle extended into the period two to six years and for the time point six years child A was coded to have been exposed to 30 months of paternal labour migration. Child B’s father left for labour migration shortly after his/her conception and had been absent for eight months by the time he/she was born. This cycle of migration ended between birth and six months and child B was coded to have been exposed to 12 months of paternal labour migration when it was six months old. For the following time points (one, two and six years) child B was coded as not having been exposed to paternal labour migration. Child C’s father left for the first time between six and 12 months and by the time she/he was one year child C had been exposed to four months of labour migration. This migration cycle only had a total duration of 14 months and ended before the second birthday, so child C was coded to have been exposed to 14 months of paternal labour migration in the period preceding two years (i.e., from one to two years). Between two and four years, child C’s father left twice to work abroad. The first cycle of migration had a total duration of 24 months, the second was still ongoing by the time the child was six years and he had been absent for 12 months at the time. For the time point six years, child C was coded to have been exposed to paternal labour migration a total of 36 months. Panel B illustrates how these three children would have been coded in a data frame.


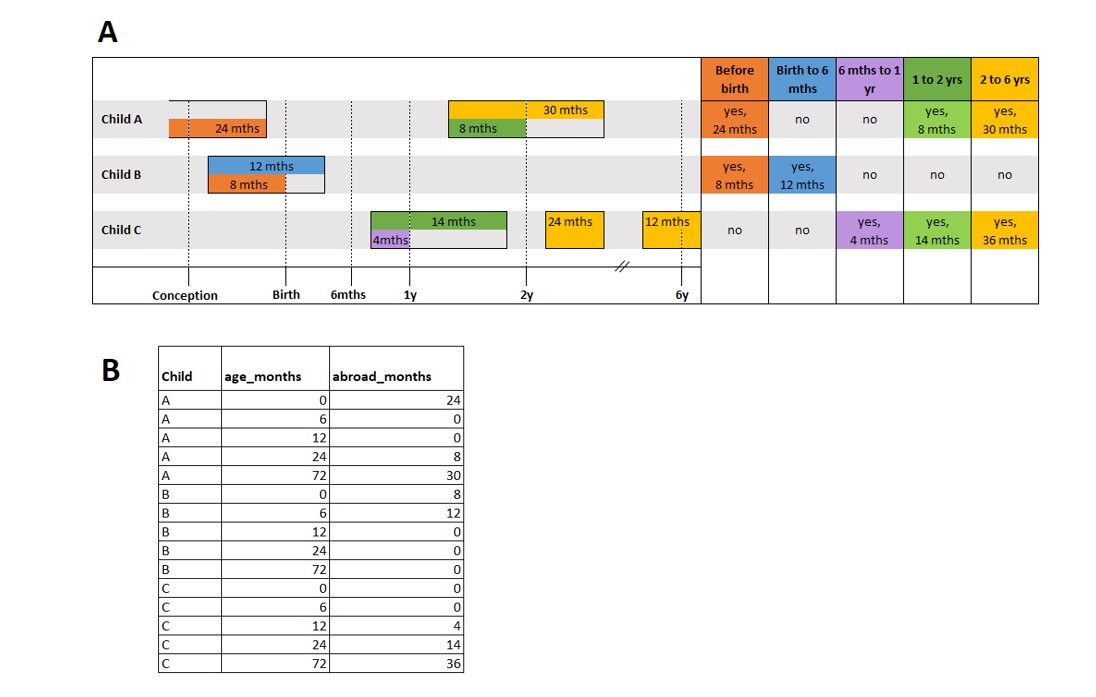


Supplementary Figure 2.1: Examples of fathers’ migration trajectories relative to the children’s age and their operationalisation into categories of exposure. Panel A visualises the paternal migration trajectories of three hypothetical examples in a timeline and demonstrates how these were summarised to convey the child’s exposure to migration. Panel B illustrates how these three children would have been coded in a data frame.

## Categorising duration of exposure to father’s migration

We decided to use a categorical variable of father’s duration of migration rather than a continuous variable because this allowed us to include one category for non-migrant fathers. In order to address research question 2, we differentiated between short- and long-term migrant fathers. To select appropriate cut-off points we first plotted the duration of the father’s stay abroad against the child’s HAZ (using the loess smoother (Cleveland, Grosse, & Shyu, 1992) to see whether we could detect a pattern. We tried different cut-off points in terms of father’s duration abroad and chose the one that had the best model fit in terms of BIC.

# Selection of confounders

## Directed Acyclic Graph (DAG) of the relationship between paternal migration, child growth, and potential confounding factors

**RQs 1-4 – Association between paternal migration and child growth:** As migration does not happen at random but is a very deliberate decision, we identified factors that may influence a father’s decision to go abroad and that are also associated with child growth. The decision to migrate may depend on the potential migrant’s ability and need to seek an income abroad. The poorest and uneducated might not be able to secure a migration loan, while better-off households with existing cash income may feel less need to send family members overseas. Food insecurity could drive a household’s decision to send one of their males abroad and whether any household member other than the father is already a migrant may influence the decision. On the one hand, migrant networks have been discussed in the literature to lower the costs of migration and make it easier to also seek work away from home (Haug, 2008). On the other hand, the remittances sent by another household member could lower the financial pressure on the father to also leave his family. Supplementary Figure 3.1 visualises our assumptions of the potential confounding factors.


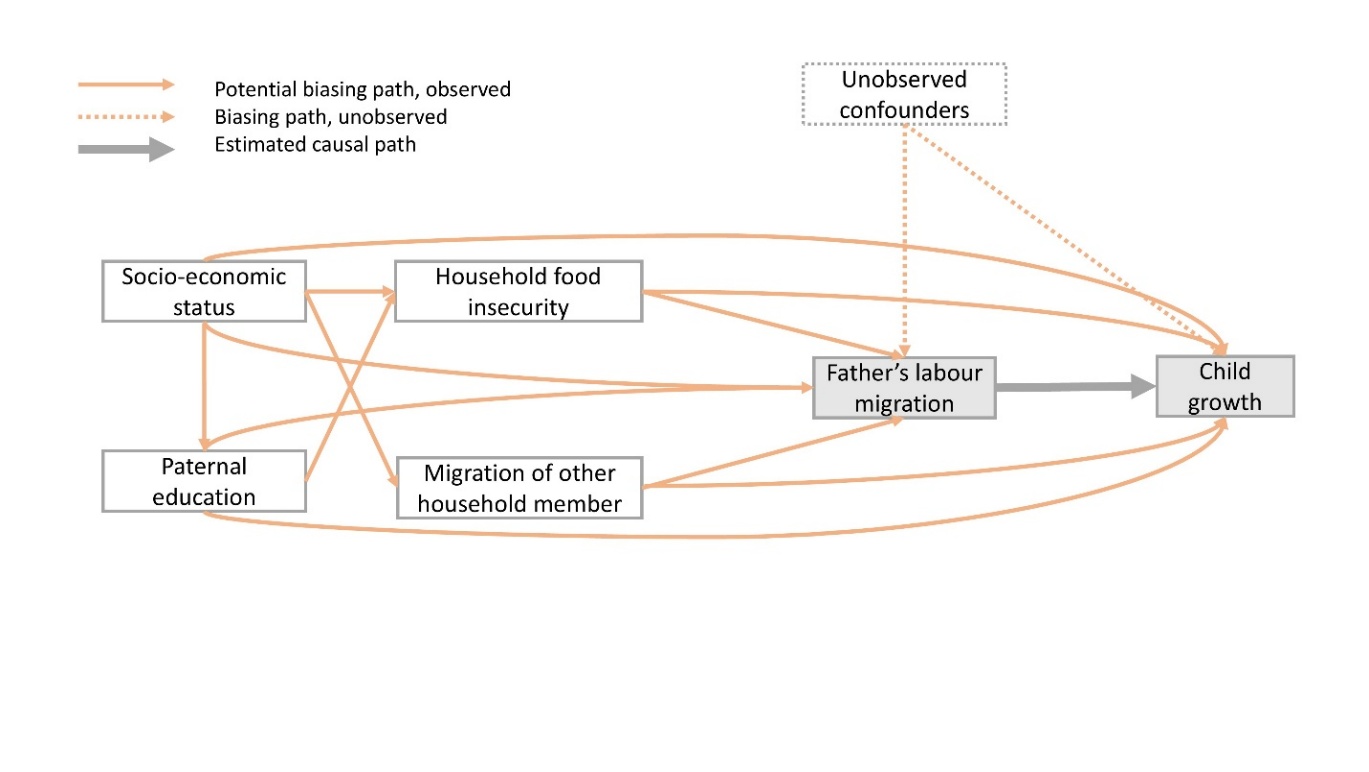
**Supplementary Figure 3.1: Directed Acyclic Graph (DAG) of the relationship between paternal migration, child growth, and potential confounding factors.**

## Directed Acyclic Graph (DAG) of the relationship between remittances, child growth, and potential confounding factors

**RQ 5 – Association between father’s net remittances and child growth**

The remittances depend among other things on the destination country, the migrant’s job, salary and additional benefits such as employer-provided housing. A potential migrant with higher education and better financial resources is likely able to access trustworthy brokers and negotiate a better contract. If another member of the household is already overseas or has experience from past migration stints, he will be able to lower the costs of migration for the father by sharing information on good job opportunities, cheap remittance channels, and economical living overseas. These potentially confounding pathways are illustrated in Supplementary Figure 3.2.

**
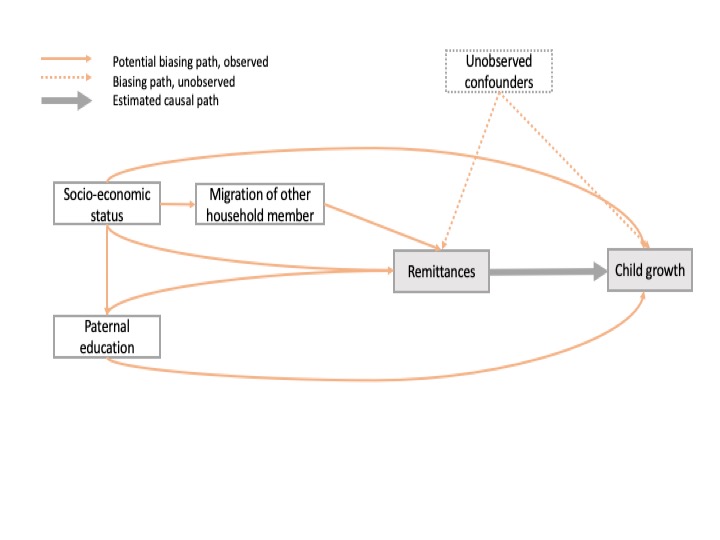
**

Supplementary Figure 3.2: Directed Acyclic Graph (DAG) of the relationship between remittances, child growth, and potential confounding factors.

# Regression results (RQs 1-3)

## Regression results (adjusted) (RQs 1-3)

Supplementary Table 4.1 shows the regression coefficients as presented in Figure 2 in the Main Text.

Supplementary Table 4.1: Results (adjusted) from mixed-effects regression to estimate the impact of father’s migration and the duration of his stay abroad on child height-for-age *z*-score, at 0-72 months, 0-6 months, and 12-72 months.^1^

|  | **0-72 months^2^** | |  |  | **0-6 months^3^** | |  |  | **12-72 months^4^** | |  |
| --- | --- | --- | --- | --- | --- | --- | --- | --- | --- | --- | --- |
|  | Coef | 95% CI | *p* |  | Coef | 95% CI | *p* |  | Coef | 95% CI | *p* |
| Father is not a migrant | Ref |  |  |  | Ref |  |  |  | Ref |  |  |
| Father is a migrant | -0.08 | ( -0.15, 0) | 0.05 |  | -0.16 | ( -0.30, -0.01) | 0.02 |  | -0.05 | ( -0.14, 0.03) | 0.26 |
|  |  |  |  |  |  |  |  |  |  |  |  |
| Father is not a migrant | Ref |  |  |  | Ref |  |  |  | Ref |  |  |
| Father has been away ≤12 months | -0.12 | ( -0.21, -0.04) | 0.01 |  | -0.23 | ( -0.4, -0.05) | 0.01 |  | -0.10 | ( -0.20, -0.01) | 0.03 |
| Father has been away >1 year | -0.03 | ( -0.12, 0.06) | 0.55 |  | -0.10 | ( -0.27, 0.07) | 0.24 |  | 0.00 | ( -0.10, 0.10) | 0.99 |

^1^ Mixed-effects regression adjusted for asset quartile, father’s education, household food insecurity, maternal height, other household member’s migration.

^2^ Random effects on both intercept and slope. 2562 observations of 525 children.

^3^ Random effects on intercept only. 1035 observations of 524 children

^4^ Random effects on both intercept and slope. 1527 observations of 525 children

## Regression results (unadjusted) (RQs 1-3)

Supplementary Table 4.2: Results (unadjusted) from mixed-effects regression to estimate the impact of father’s migration and the duration of his stay abroad on child height-for-age *z*-score, at 0-72 months, 0-6 months, and 12-72 months.

|  | **0-72 months^1^** | |  |  | **0-6 months^2^** | |  |  | **12-72 months^3^** | |  |
| --- | --- | --- | --- | --- | --- | --- | --- | --- | --- | --- | --- |
|  | Coef | 95% CI | *p* |  | Coef | 95% CI | *p* |  | Coef | 95% CI | *p* |
| Father is not a migrant | Ref |  |  |  | Ref |  |  |  | Ref |  |  |
| Father is a migrant | -0.08 | ( -0.16, 0) | 0.05 |  | -0.15 | ( -0.30, -0.01) | 0.02 |  | -0.05 | ( -0.13, 0.04) | 0.26 |
|  |  |  |  |  |  |  |  |  |  |  |  |
| Father is not a migrant | Ref |  |  |  | Ref |  |  |  | Ref |  |  |
| Father has been away ≤12 months | -0.12 | ( -0.21, -0.03) | 0.01 |  | -0.23 | ( -0.40, -0.05) | 0.01 |  | -0.10 | ( -0.20, -0.01) | 0.03 |
| Father has been away >1 year | -0.03 | ( -0.12, 0.06) | 0.52 |  | -0.10 | ( -0.27, 0.07) | 0.24 |  | 0.00 | ( -0.10, 0.10) | 0.99 |

^1^ Random effects on both intercept and slope. 2562 observations of 525 children.

^2^ Random effects on intercept only. 1035 observations of 524 children

^3^ Random effects on both intercept and slope. 1527 observations of 525 children

## Regression results (sex-stratified) (RQs 1-3)

Supplementary Table 4.3.1: Results from mixed-effects regression to estimate the impact of father’s migration and the duration of his stay abroad on child height-for-age *z*-score, at 0-72 months, 0-6 months, and 12-72 months. Results for girls only.

|  | **0-72 months^1^** | |  |  | **0-6 months^2^** | |  |  | **12-72 months^3^** | |  |
| --- | --- | --- | --- | --- | --- | --- | --- | --- | --- | --- | --- |
|  | Coef | 95% CI | *p* |  | Coef | 95% CI | *p* |  | Coef | 95% CI | *p* |
| Father is not a migrant | Ref |  |  |  | Ref |  |  |  | Ref |  |  |
| Father is a migrant | -0.13 | ( -0.24, -0.03) | 0.01 |  | -0.23 | ( -0.41, -0.05) | 0.01 |  | -0.09 | ( -0.21, 0.03) | 0.15 |
|  |  |  |  |  |  |  |  |  |  |  |  |
| Father is not a migrant | Ref |  |  |  | Ref |  |  |  | Ref |  |  |
| Father has been away ≤12 months | -0.17 | ( -0.28, -0.05) | 0 |  | -0.22 | ( -0.45, 0.01) | 0.06 |  | -0.16 | ( -0.29, -0.03) | 0.02 |
| Father has been away >1 year | -0.09 | ( -0.21, 0.04) | 0.17 |  | -0.24 | ( -0.46, -0.01) | 0.04 |  | 0.00 | ( -0.14, 0.14) | 1 |

^1^ Random effects on both intercept and slope. 1225 observations of 250 children.

^2^ Random effects on intercept only. 497 observations of 250 children

^3^ Random effects on both intercept and slope. 728 observations of 250 children

Supplementary Table 4.3.2: Results from mixed-effects regression to estimate the impact of father’s migration and the duration of his stay abroad on child height-for-age *z*-score, at 0-72 months, 0-6 months, and 12-72 months. Results for boys only.

|  | **0-72 months^1^** | |  |  | **0-6 months^2^** | |  |  | **12-72 months^3^** | |  |
| --- | --- | --- | --- | --- | --- | --- | --- | --- | --- | --- | --- |
|  | Coef | 95% CI | *p* |  | Coef | 95% CI | *p* |  | Coef | 95% CI | *p* |
| Father is not a migrant | Ref |  |  |  | Ref |  |  |  | Ref |  |  |
| Father is a migrant | -0.03 | ( -0.14, 0.08) | 0.63 |  | -0.11 | ( -0.31, 0.1) | 0.32 |  | -0.01 | ( -0.13, 0.1) | 0.82 |
|  |  |  |  |  |  |  |  |  |  |  |  |
| Father is not a migrant | Ref |  |  |  | Ref |  |  |  | Ref |  |  |
| Father has been away ≤12 months | -0.09 | ( -0.21, 0.04) | 0.19 |  | -0.26 | ( -0.51, 0.0) | 0.05 |  | -0.05 | ( -0.19, 0.09) | 0.49 |
| Father has been away >1 year | 0.03 | ( -0.1, 0.15) | 0.67 |  | 0.04 | ( -0.21, 0.29) | 0.75 |  | 0.02 | ( -0.12, 0.15) | 0.82 |

^1^ Random effects on both intercept and slope. 1337 observations of 275 children.

^2^ Random effects on intercept only. 538 observations of 274 children

^3^ Random effects on both intercept and slope. 799 observations of 275 children

# Results of other outcomes at six years (RQ 4)

## 5.1 Coefficient plots on the association between fathers’ migration and other measures of child growth at six years

Supplementary Figure 5.1: Association between father's migration and child skinfold thickness at six years.

Supplementary Figure 5.2: Association between father's migration and child body composition at six years.

**Supplementary Figure 5.3: Association between father's migration and the child's tibia length and grip strength category at six years.**

## Results tables on the association between fathers’ migration and other measures of child growth at six years

Supplementary Table 5.1 shows the regression coefficients as presented in Figure 3 in the Main Text.

Supplementary Table 5.1: Association between father’s migration for work and left-behind children’s body circumferences at six years. Results from linear regressions. Adjusted for migration of other household member, maternal height, asset quartile, husband’s education.

|  | **Head** |  |  | **MUAC** |  |  | **Waist** |  |  | **Hip** |  |  | **Calf** |  |  |
| --- | --- | --- | --- | --- | --- | --- | --- | --- | --- | --- | --- | --- | --- | --- | --- |
|  | Coef | SE | *p* | Coef | SE | *p* | Coef | SE | *p* | Coef | SE | *p* | Coef | SE | *p* |
| Father never migrated | Ref |  |  | Ref |  |  | Ref |  |  | Ref |  |  | Ref |  |  |
| Migration at any time in child's life | -0.23 | 0.12 | 0.06 | -0.23 | 0.11 | 0.04 | -0.58 | 0.26 | 0.03 | -0.44 | 0.29 | 0.13 | -0.3 | 0.13 | 0.02 |
| Father did not migrate before childbirth | Ref |  |  | Ref |  |  | Ref |  |  | Ref |  |  | Ref |  |  |
| Migration before birth | -0.31 | 0.12 | 0.01 | -0.15 | 0.11 | 0.17 | -0.2 | 0.26 | 0.44 | -0.38 | 0.29 | 0.18 | -0.15 | 0.13 | 0.25 |
| Father did not migrate at age birth to 6mths | Ref |  |  | Ref |  |  | Ref |  |  | Ref |  |  | Ref |  |  |
| Migration at birth to 6mths | -0.34 | 0.12 | 0 | -0.14 | 0.11 | 0.19 | -0.36 | 0.26 | 0.16 | -0.57 | 0.28 | 0.05 | -0.32 | 0.13 | 0.01 |
| Father did not migrate age 6mths to 1y | Ref |  |  | Ref |  |  | Ref |  |  | Ref |  |  | Ref |  |  |
| Migration at 6mths to 1y | -0.25 | 0.11 | 0.02 | -0.19 | 0.1 | 0.06 | -0.45 | 0.25 | 0.07 | -0.58 | 0.27 | 0.03 | -0.32 | 0.12 | 0.01 |
| Father did not migrate age 1-2y | Ref |  |  | Ref |  |  | Ref |  |  | Ref |  |  | Ref |  |  |
| Migration at 1-2y | -0.14 | 0.11 | 0.2 | -0.07 | 0.1 | 0.49 | -0.41 | 0.24 | 0.09 | -0.34 | 0.27 | 0.21 | -0.21 | 0.12 | 0.08 |
| Father did not migrate age 2-6y | Ref |  |  | Ref |  |  | Ref |  |  | Ref |  |  | Ref |  |  |
| Migration at 2-6y | -0.24 | 0.12 | 0.04 | -0.21 | 0.11 | 0.05 | -0.53 | 0.25 | 0.04 | -0.37 | 0.28 | 0.19 | -0.28 | 0.13 | 0.03 |
| *n* | 523 |  |  | 524 |  |  | 524 |  |  | 524 |  |  | 524 |  |  |

Supplementary Table 5.2 shows the regression coefficients as presented in Supplementary Figure 5.1.

Supplementary Table 5.2: Association between father’s migration for work and left-behind children’s skinfold thickness at six years. Results from linear regressions. Adjusted for migration of other household member, maternal height, asset quartile, husband’s education, household food insecurity.

|  | **Biceps** |  |  | **Triceps** |  |  | **Subscapular** |  |  | **Suprailiac** |  |  |
| --- | --- | --- | --- | --- | --- | --- | --- | --- | --- | --- | --- | --- |
|  | Coef | SE | *p* | Coef | SE | *p* | Coef | SE | *p* | Coef | SE | *p* |
| Father never migrated | Ref |  |  | Ref |  |  | Ref |  |  | Ref |  |  |
| Migration at any time in child's life | -0.14 | 0.1 | 0.18 | -0.25 | 0.14 | 0.09 | -0.25 | 0.09 | 0.01 | -0.35 | 0.16 | 0.03 |
| Father did not migrate before childbirth | Ref |  |  | Ref |  |  | Ref |  |  | Ref |  |  |
| Migration before birth | -0.11 | 0.1 | 0.28 | -0.13 | 0.14 | 0.38 | -0.08 | 0.09 | 0.4 | -0.21 | 0.16 | 0.18 |
| Father did not migrate at age birth to 6mths | Ref |  |  | Ref |  |  | Ref |  |  | Ref |  |  |
| Migration at birth to 6mths | -0.06 | 0.1 | 0.55 | 0.01 | 0.14 | 0.95 | -0.08 | 0.09 | 0.38 | -0.15 | 0.16 | 0.32 |
| Father did not migrate age 6mths to 1y | Ref |  |  | Ref |  |  | Ref |  |  | Ref |  |  |
| Migration at 6mths to 1y | -0.07 | 0.1 | 0.44 | -0.02 | 0.14 | 0.88 | -0.05 | 0.09 | 0.55 | -0.15 | 0.15 | 0.31 |
| Father did not migrate age 1-2y | Ref |  |  | Ref |  |  | Ref |  |  | Ref |  |  |
| Migration at 1-2y | -0.04 | 0.1 | 0.67 | -0.09 | 0.14 | 0.5 | -0.06 | 0.08 | 0.45 | -0.11 | 0.15 | 0.46 |
| Father did not migrate age 2-6y | Ref |  |  | Ref |  |  | Ref |  |  | Ref |  |  |
| Migration at 2-6y | -0.15 | 0.1 | 0.14 | -0.19 | 0.14 | 0.17 | -0.26 | 0.09 | 0 | -0.29 | 0.15 | 0.06 |
| *n* | 523 |  |  | 522 |  |  | 520 |  |  | 523 |  |  |

Supplementary Table 5.3 shows the regression coefficients as presented in Supplementary Figure 5.2.

Supplementary Table 5.3: Association between father’s migration for work and left-behind children’s lean mass at six years. Results from linear regressions. Adjusted for migration of other household member, maternal height, asset quartile, husband’s education, household food insecurity.

|  | **Lean mass** |  |  | **Lean mass index** |  |  | **Lean mass z-score** |  |  |
| --- | --- | --- | --- | --- | --- | --- | --- | --- | --- |
|  | Coef | SE | *p* | Coef | SE | *p* | Coef | SE | *p* |
| Father never migrated | Ref |  |  | Ref |  |  | Ref |  |  |
| Migration at any time in child's life | -0.42 | 0.16 | 0.01 | -0.16 | 0.1 | 0.09 | -0.17 | 0.09 | 0.05 |
| Father did not migrate before childbirth | Ref |  |  | Ref |  |  | Ref |  |  |
| Migration before birth | -0.11 | 0.16 | 0.51 | 0.01 | 0.1 | 0.93 | -0.06 | 0.09 | 0.53 |
| Father did not migrate at age birth to 6mths | Ref |  |  | Ref |  |  | Ref |  |  |
| Migration at birth to 6mths | -0.4 | 0.16 | 0.01 | -0.16 | 0.09 | 0.09 | -0.23 | 0.09 | 0.01 |
| Father did not migrate age 6mths to 1y | Ref |  |  | Ref |  |  | Ref |  |  |
| Migration at 6mths to 1y | -0.42 | 0.15 | 0.01 | -0.19 | 0.09 | 0.04 | -0.22 | 0.08 | 0.01 |
| Father did not migrate age 1-2y | Ref |  |  | Ref |  |  | Ref |  |  |
| Migration at 1-2y | -0.27 | 0.15 | 0.08 | -0.16 | 0.09 | 0.08 | -0.14 | 0.08 | 0.09 |
| Father did not migrate age 2-6y | Ref |  |  | Ref |  |  | Ref |  |  |
| Migration at 2-6y | -0.36 | 0.16 | 0.02 | -0.14 | 0.09 | 0.14 | -0.15 | 0.09 | 0.08 |
| *n* | 471 |  |  | 471 |  |  | 471 |  |  |

Supplementary Table 5.4 shows the regression coefficients as presented in Supplementary Figure 5.3.

Supplementary table 5.4: Association between father’s migration for work and left-behind children’s fat mass at six years. Results from linear regressions. Adjusted for migration of other household member, maternal height, asset quartile, husband’s education, household food insecurity.

|  | **Fat mass** |  |  | **Fat mass index** |  |  | **Fat mass z-score** |  |  |
| --- | --- | --- | --- | --- | --- | --- | --- | --- | --- |
|  | Coef | SE | *p* | Coef | SE | *p* | Coef | SE | *p* |
| Father never migrated | Ref |  |  | Ref |  |  | Ref |  |  |
| Migration at any time in child's life | -0.11 | 0.11 | 0.3 | -0.06 | 0.08 | 0.48 | -0.07 | 0.12 | 0.6 |
| Father did not migrate before childbirth | Ref |  |  | Ref |  |  | Ref |  |  |
| Migration before birth | -0.24 | 0.11 | 0.02 | -0.19 | 0.08 | 0.02 | -0.21 | 0.12 | 0.08 |
| Father did not migrate at age birth to 6mths | Ref |  |  | Ref |  |  | Ref |  |  |
| Migration at birth to 6mths | -0.1 | 0.1 | 0.32 | -0.06 | 0.08 | 0.49 | -0.03 | 0.12 | 0.84 |
| Father did not migrate age 6mths to 1y | Ref |  |  | Ref |  |  | Ref |  |  |
| Migration at 6mths to 1y | -0.07 | 0.1 | 0.49 | -0.03 | 0.08 | 0.72 | 0 | 0.12 | 0.97 |
| Father did not migrate age 1-2y | Ref |  |  | Ref |  |  | Ref |  |  |
| Migration at 1-2y | -0.04 | 0.1 | 0.72 | -0.02 | 0.08 | 0.76 | 0.01 | 0.12 | 0.95 |
| Father did not migrate age 2-6y | Ref |  |  | Ref |  |  | Ref |  |  |
| Migration at 2-6y | -0.12 | 0.1 | 0.25 | -0.07 | 0.08 | 0.4 | -0.09 | 0.12 | 0.47 |
| *n* | 471 |  |  | 471 |  |  | 471 |  |  |

Supplementary Table 5.5 shows the regression coefficients as presented in Supplementary Figure 5.4.

Supplementary table 5.5: Association between father’s migration for work and left-behind children’s tibia length and grip strength category at six years. Results from linear regression (tibia length) and ordinal logistic regression (grip strength category). Adjusted for migration of other household member, maternal height, asset quartile, husband’s education, household food insecurity.

|  | **Tibia length** |  |  | **Grip strength category** |  |
| --- | --- | --- | --- | --- | --- |
|  | Coef | SE | *p* | OR | 95% CI |
| Father never migrated | Ref |  |  | Ref |  |
| Migration at any time in child's life | -1.24 | 1.29 | 0.34 | 0.85 | 0.6 |
| Father did not migrate before childbirth | Ref |  |  | Ref |  |
| Migration before birth | 0.57 | 1.28 | 0.66 | 0.95 | 0.68 |
| Father did not migrate at age birth to 6mths | Ref |  |  | Ref |  |
| Migration at birth to 6mths | -0.34 | 1.26 | 0.79 | 1.05 | 0.75 |
| Father did not migrate age 6mths to 1y | Ref |  |  | Ref |  |
| Migration at 6mths to 1y | -0.31 | 1.21 | 0.8 | 1.07 | 0.77 |
| Father did not migrate age 1-2y | Ref |  |  | Ref |  |
| Migration at 1-2y | 0.39 | 1.2 | 0.75 | 1.06 | 0.77 |
| Father did not migrate age 2-6y | Ref |  |  | Ref |  |
| Migration at 2-6y | -1.32 | 1.25 | 0.29 | 0.8 | 0.57 |
| *n* | 514 |  |  | 523 |  |

# Coefficient plots on the association between father’s net remittances and child growth outcomes (RQ 5)

Supplementary Figure 6: Father’s net remittances (in 100k NPR) and child growth outcomes at six years. Results from linear regressions and ordinal logistic regression (for outcome grip strength). Adjusted for migration of other household member, maternal height, asset quartile, father’s education.
